# Opsin Repertoire and Expression Patterns in Horseshoe Crabs: Evidence from the Genome of *Limulus polyphemus* (Arthropoda: Chelicerata)

**DOI:** 10.1093/gbe/evw100

**Published:** 2016-04-29

**Authors:** Barbara-Anne Battelle, Joseph F. Ryan, Karen E. Kempler, Spencer R. Saraf, Catherine E. Marten, Wesley C. Warren, Patrick J. Minx, Michael J. Montague, Pamela J. Green, Skye A. Schmidt, Lucinda Fulton, Nipam H. Patel, Meredith E. Protas, Richard K. Wilson, Megan L. Porter

**Affiliations:** ^1^Whitney Laboratory for Marine Bioscience, Departments of Neuroscience and Biology, University of Florida; ^2^Whitney Laboratory for Marine Bioscience, Department of Biology, University of Florida; ^3^McDonnell Genome Institute, Washington University School of Medicine in St. Louis; ^4^Department of Plant and Soil Sciences, School of Marine Science and Policy, Delaware Biotechnology Institute, University of Delaware; ^5^Department of Molecular Cell Biology, Center for Integrative Genomics, University of California, Berkley; ^6^Department of Biology, University of Hawaii, Manoa; ^7^Present address: School of Marine and Atmospheric Sciences, Stony Brook University, Stony Brook, NY; ^8^Present address: Department of Molecular Genetics and Microbiology, College of Medicine, University of Florida, Gainesville, FL; ^9^Present address: Department of Natural Sciences and Mathematics, Dominican University of California, San Rafael, CA

**Keywords:** Limulus polyphemus, xiphosuran, opsin, photoreceptors, evolution

## Abstract

Horseshoe crabs are xiphosuran chelicerates, the sister group to arachnids. As such, they are important for understanding the most recent common ancestor of Euchelicerata and the evolution and diversification of Arthropoda. *Limulus polyphemus* is the most investigated of the four extant species of horseshoe crabs, and the structure and function of its visual system have long been a major focus of studies critical for understanding the evolution of visual systems in arthropods. Likewise, studies of genes encoding *Limulus* opsins, the protein component of the visual pigments, are critical for understanding opsin evolution and diversification among chelicerates, where knowledge of opsins is limited, and more broadly among arthropods. In the present study, we sequenced and assembled a high quality nuclear genomic sequence of *L. polyphemus* and used these data to annotate the full repertoire of *Limulus* opsins. We conducted a detailed phylogenetic analysis of *Limulus* opsins, including using gene structure and synteny information to identify relationships among different opsin classes. We used our phylogeny to identify significant genomic events that shaped opsin evolution and therefore the visual system of *Limulus*. We also describe the tissue expression patterns of the 18 opsins identified and show that transcripts encoding a number, including a peropsin, are present throughout the central nervous system. In addition to significantly extending our understanding of photosensitivity in *Limulus* and providing critical insight into the genomic evolution of horseshoe crab opsins, this work provides a valuable genomic resource for addressing myriad questions related to xiphosuran physiology and arthropod evolution.

## Introduction

The American horseshoe crab *Limulus polyphemus* (Linnaeus 1758) is one of four extant species of xiphosuran chelicerates, the sister group to arachnids (Regier et al. 2010; Edgecombe and Legg 2014). As such, studies of horseshoe crabs are key to understanding the origin of arachnids and the most recent common ancestor of Euchelicerata. In addition, the Euchelicerata ancestor is a key node for better understanding the evolution of arthropods in general. *Limulus polyphemus*, hereafter referred to as *Limulus*, is the most studied of the extant horseshoe crabs, and investigations of its visual system have been central to understanding basic mechanisms of vision including phototransduction (e.g., Brown et al. 1984; Shin et al. 1993), light- and dark-adaptation (e.g., Lisman and Brown 1972; Behrens and Krebs 1976), visual information processing (Hartline et al. 1956) and the effects of circadian rhythms on visual function (Battelle 2013). Investigations of the *Limulus* visual system may also provide insights into the organization and function of visual systems in the most recent common ancestor of Arthropoda (Nilsson and Kelber 2007). Likewise, studies of genes encoding *Limulus* opsins, the protein component of the visual pigment, are central to understanding opsin evolution and diversification among chelicerates, a group in which knowledge of opsin proteins is limited.

Opsins have been classified into four major monophyletic groups: rhabdomeric or R-type opsins such as those found in the microvillar-rich photoreceptors in the eyes of arthropods, ciliary or C-type opsins such as those found in the ciliary rods and cones of vertebrates, cnidarians opsins or Cnidops, which appear unique to cnidarians, and retinal G-protein-coupled receptors (RGR)/Go-type or Group 4 opsins consisting of a mixed group of RGR, peropsins and neuropsins (reviewed in Porter et al. 2012). In previous studies, we determined that most photoreceptors in *Limulus* eyes express more than one R-type opsin.

**Fig. 1.—:**
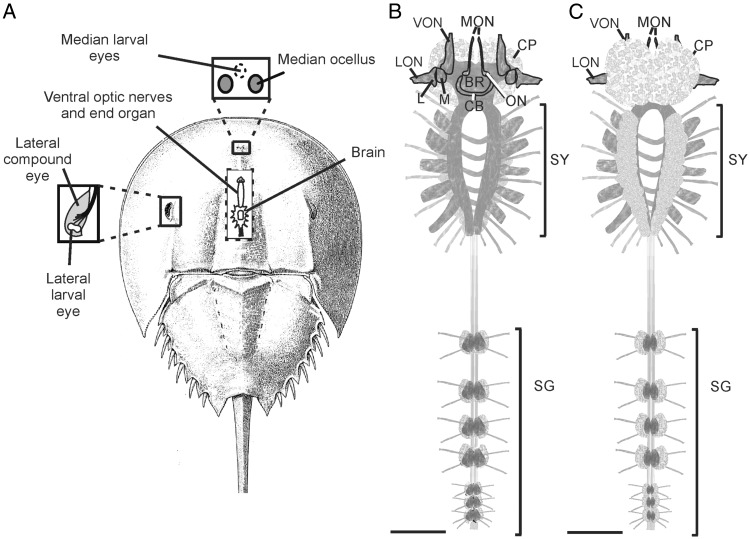
Schematics showing the locations of *Limulus* eyes and the structure of its CNS. (*A*) Dorsal view of an adult animal showing the locations of its eyes. The upper box is an enlargement showing the median ocelli and the fused median larval eyes between them. The box on the left shows an enlargement of a lateral compound eye and the location of the lateral larval eye at its posterior edge. The cut-away in the center shows the locations of the brain and ventral optic nerves projecting from the brain to the end organ. The synganglion posterior to the brain is also shown. (*B*) Dorsal and (*C*) ventral view of the CNS of a juvenile animal measuring ∼2–2.5 cm across the prosoma. BR, brain; CB, central body; CP, corpora pedunculata; L, lamina; LON, lateral optic nerve; M, medulla; MON, median optic nerve; ON, ocellar neuropile; SG, segmental ganglia (abdominal ganglia); SY, synganglion (circumesophageal ring); VON, ventral optic nerve. Scale bar, 1 mm.

*Limulus* has three different types of eyes: a pair of lateral compound, image-forming eyes called lateral eyes (LE), a pair of median ocelli called median eyes (ME) and three pair of larval eyes, lateral, median and ventral (fig. 1*A*). Among the larval eyes, the ventral larval eyes or ventral eyes (VE) have been studied most extensively. Five opsin genes—LpOps1–4, which encode nearly identical transcripts and therefore are considered a set (Dalal et al. 2003), and LpOps5—are co-expressed in LE retinular cells and giant photoreceptors in larval eyes (Katti et al. 2010), two opsins (LpOps5 and LpUVOps1) are co-expressed in small photoreceptors in larval eyes (Battelle et al. 2014), and three opsins (LpOps6, 7, and 8) are co-expressed in visible light sensitive photoreceptors in MEs (Battelle et al. 2015). We determined that in addition to being expressed in UV-sensitive ME photoreceptors, LpUVOps1 is expressed in LE eccentric cells (Battelle et al. 2014), a cell type originally thought to be a nonphotosensitive secondary cell (Waterman and Wiersma 1954). We showed further that a peropsin, LpPerOps1, is expressed in glia or pigment cells surrounding photoreceptors in each of the eyes (Battelle et al. 2015).

To aid in further studies of xiphosuran chelicerate visual systems in general, and of *Limulus* opsin genes in particular, we generated a high-quality genome assembly of *L. polyphemus*. Two previous studies have published genome sequences for *Limulus*, however the genome is large and resulting assemblies suffer from very low contiguity (Nossa et al. 2014; Kenny et al. 2016). The N50 values for these assemblies were all under 3 kb, which limits the types of analyses that can be performed. For example, large genes can be scattered across multiple scaffolds making it impossible to study gene structure, and it is impossible to study extended synteny on at least 50% of the genome. For these reasons, these sub-draft assemblies are not useful for an in-depth analysis of an extended gene family like the opsins. Using our high-quality genome assembly, we characterized the full repertoire of opsins in *Limulus*, conducted detailed phylogenetic analyses to classify each *Limulus* opsin, and used gene structure and synteny information to verify classifications and identify interesting genomic events that likely shaped the evolution of arthropod visual systems. Lastly, we provide detailed information on the tissue expression patterns for each of the 18 *Limulus* opsins, making this animal a pivotal resource for understanding the evolution of opsins and photosensitivity.

## Materials and Methods

### Reagents

Unless otherwise specified, reagents were purchased from Fisher Scientific (Pittsburgh, PA) or Sigma–Aldrich (St. Louis, MO).

### Genome Sequencing

Genetic material used for sequencing was obtained during January 2008 from a single adult male (carapace length: ∼16 cm) purchased from the Marine Biological Laboratory (Woods Hole, MA). Genomic DNA was prepared from limb muscle tissue, and source DNA (UCB-LP #5) is available from the lab of Nipam Patel at the University of California, Berkeley, CA.

We generated 18× sequence coverage (fragments, 3 and 8 kb) with reads generated on Roche 454 instrumentation. These combined sequence reads were assembled using the Newbler software (Roche Sequencing, Pleasanton, CA, a division of Hoffmann-La Roche LTD,Basel, Switzerland), and where possible, scaffold gaps were closed by mapping with 12× coverage of Illumina sequences and local gap assembly.

### Animals for Opsin Experiments

Adult *Limulus* were collected from the Indian River near Melbourne, FL (Latitude 28°42′ 31.46″ N; Longitude 80°44′ 53.06″ W). Young juveniles, between their first and second juvenile molts, were reared at the Whitney Lab following *in vitro* fertilization using eggs and sperm from adults also collected from the Indian River. Older juveniles, measuring 2.5–3.5 cm across the prosoma, were purchased from Pet Solutions (Bevercreek, OH). Adult animals were maintained in naturally running seawater held between 18 °C and 20 °C and fed shrimp twice a week. Juveniles were maintained in large containers of shallow, natural sea water over sandy bottoms. Twice a week, the seawater was changed and the juveniles were fed *Artemia*. All animals were maintained under natural illumination provided by a skylight in the aquarium room. Natural light intensities in the aquarium room were monitored continuously using a HOBO light data logger (Onset Computer Corporation, Pocasset, MA). They peaked midday at ∼70,000 lux. The spectrum of light was also measured from 300–850 nm using an Ocean Optics USB4000 UV-visible spectrometer fitted with a 200-µm diameter UV-visible fiber (Ocean Optics, Dunedin, FL). No light with wavelengths <400 nm penetrated the skylight.

### RNA Isolations and cDNA Preparation

We used RNeasy (Qiagen, Valencia, CA) to isolate RNA from the following tissues from older juveniles: brain, tail and the synganglion pooled with all segmental ganglia. We also used RNeasy to isolate RNA from adult MEs and VEs. We used RNAzol to isolate RNA from adult LE, brain, synganglia and all segmental ganglia pooled together. To prepare RNA from large juveniles, we pooled tissues from three or four animals. To prepare RNA from adults, we used one LE, eight MEs, eight VEs, one or two brains, one synganglion and all segmental ganglia pooled from a single animal. In some instances, we removed a portion of the brain anterior to the optic ganglia to reduce contamination by ventral photoreceptors attached to the brain. RNA was reverse transcribed with SuperScript III-First Strand Synthesis System for RT-PCR (Life Technologies, Grand Island, NY). The cDNA library was prepared from RNA isolated from the entire CNS of young juveniles (Katti et al. 2010). All animals were sacrificed and RNA was extracted in the morning in the light.

### Opsin Cloning

The following opsins were cloned and characterized previously: LpOps1 and 2 (Smith et al. 1993), LpOps5 (Katti et al. 2010), LpUVOps1 (Battelle et al. 2014), and LpOps6, 7, 8 and LpPerOps1 (Battelle et al. 2015). Previous studies also identified two additional LpOps1-like genes called LpOps3 and 4 (Dalal et al. 2003) and a second peropsin gene called LpPerOps2 (Battelle et al. 2015).

In the present study, we identified sequences encoding portions of five additional presumptive R-type opsins in a TBLASTN search of our *Limulus* genome assembly (GenBank accession: GCA_000517525.1) with LpOps1 (Accession number AAA02499) as query. As we detail below, we used PCR to amplify putative opsins from cDNA prepared from various *Limulus* tissues and cloned partial or full-length open reading frames (ORF) into pGem-T (Promega Corp. Madison, WI). The primers we used are listed in supplementary table S1, Supplementary Material online.

We identified LpUVOps2 as a 650-base pair (bp) opsin-like genomic sequence and amplified cDNA encoded by this sequence from adult VE and CNS using primers UVOps2F1 and UVOps2R1. We obtained its full-length ORF using a RACE (Rapid Amplification of cDNA Ends) strategy (Katti et al. 2010) with the cDNA library from young juvenile CNS as template (5′ RACE primers: UVOps2R1 and CAP followed by UVOps2R2 and CAP; 3′ RACE primers UVOps2F1 and TRSALu4 followed by UVOps2F2 and Lu4NS). We amplified the full-length ORF from adult brain cDNA with primers UVOpsF6 and UVOpsR4 (LpUVOps2 Accession number KU40433).

We identified LpOps9 as a 731-bp opsin-like genomic sequence and amplified cDNA encoded by this sequence from adult LE, VE, ME, brain, segmental ganglia and juvenile tail using primers F1 and R1. We predicted the full-length ORF from the genome assembly and amplified it from adult brain cDNA with primers F2 and R2 (LpOps9 Accession number KU40434). We identified LpOps10 as a 665-bp opsin-like genomic sequence and amplified cDNA encoded by this sequence from the young juvenile CNS cDNA library and juvenile tail cDNA using primers F1 and R1. We extended the sequence toward the 5′ and 3′ ends with primers F6 and R5 designed based on the genomic assembly and cloned the resulting 1008-bp piece (LpOps10 Accession number KU40435). We were unable to obtain a full-length clone either by RACE or by PCR using primers designed based on the genomic assembly.

We identified LpArthOps1 and LpArthOps2 as 750- and 657-bp opsin-like genomic sequences, respectively. We amplified cDNA encoded by the LpArthOps1 genomic fragment with primers F1 and R3 from adult brain and segmental ganglia cDNA. We obtained its full-length ORF with the RACE strategy described above using the cDNA library from young juvenile CNS as template. Gene-specific primers for the 5′ RACE were R5 followed by R4; for the 3′ RACE they were F1 followed by F3. We amplified the full-length ORF with primers F4 and R6 using the cDNA library from young juvenile CNS as template (LpArthOps1 Accession number KU40431). We amplified cDNA encoded by the LpArthOps2 genomic fragment with primers F1 and R3 from young juvenile CNS cDNA. We predicted its full-length ORF from the genome assembly based on its homology with LpArthOps1, and we amplified its full-length ORF from the young juvenile CNS cDNA library with primers F4 and R6 (LpArthops2 Accession number KU40432).

We additionally found portions of two presumptive *Limulus* C-type opsin genes, LpCOps1 (585 bp) and LpCOps2 (592 bp), with a TBLASTN search of the *Limulus* genome assembly using a C-type opsin amino acid sequence from a spider (Accession number CCP46950) (Eriksson et al. 2013) as query. We amplified cDNAs encoded by these genomic sequences from adult brain and segmental ganglia cDNA with LpCOps1 primers F1and R1 and LpCOps2 primers F2 and R2. Using adult brain cDNA as template, we extended the LpCOps1 sequence toward the 3′ end with primers F6 and R10 designed based on the genomic assembly and obtained an 831-bp sequence. We extended LpCOps2 toward the 5′ end with primers F12 and R4 and obtained a 740-bp sequence. We were unable to obtain full-length sequences for either C-type opsin by RACE or by PCR using primers based on gene predictions. Accession numbers of the partial sequences of LpCOps1 and 2 are KU40436 and KU40437, respectively.

### Opsin Gene Phylogenetic Analysis

In order to place the identified *Limulus* opsins within the most current understanding of opsin evolution and classification, we reconstructed a phylogeny using recent large opsin data sets (Feuda et al. 2012, 2014; Porter et al. 2012; Henze and Oakley 2015) as well as more recently published arthropod opsin data (e.g., Eriksson et al. 2013; Hering and Meyer 2014; Hwang et al. 2014). We added to these data sets several non-*Limulus* chelicerate opsin sequences including those from the spider mite *Tetranychus urticae* (Grbić et al. 2011) and those we mined from the genomes of the scorpion *Mesobuthus martensii* (Cao et al. 2013) (supplementary table S2, Supplementary Material online) and two other horseshoe crabs, *Carcinoscorpius rotundicauda* and *Tachypleus tridentatusm* (Kenny et al. 2016) (supplementary table S3, Supplementary Material online), using *Limulus* opsins as queries for TBLASTN.

Two phylogenies were reconstructed for understanding euchelicerate, and specifically *Limulus*, opsin evolution: one large data set of 743 genomic and expressed sequences representing the known evolutionary diversity of opsin proteins and one small data set consisting of all known *Limulus* opsins. Both data sets consisted of amino acid sequences aligned using MAFFT (Katoh et al. 2002; Katoh and Standley 2013). The resulting alignments were used to estimate phylogenetic relationships and node confidence as bootstrap values using RAxML (Stamatakis 2006, 2014; Liu et al. 2012 Stamatakis et al. 2008; Pattengale et al. 2009) with a GTR + G model of evolution (Feuda et al. 2014) as implemented in CIPRES (Miller et al. 2010). Both phylogenies were rooted using related GPCR melatonin receptors and/or *Trichoplax adherens* sequences as out groups as outlined in Feuda et al. (2014). For both phylogenies, opsin amino acid sequence alignments, sequence database information, and newick tree files have been deposited on DRYAD digital repository (doi:10.5061/dryad.k43t2).

Intron positions and phases of LpOps1–4 were determined previously (Dalal et al. 2003). We identified intron positions and phases of other opsins from TBLASTN analyses of the *Limulus* genome assembly and from well-assembled genomes of other species,

There were two cases where the topology of our tree suggested extraordinary evolutionary findings, whereas an alternative topology would lead to a simpler explanation. The simpler alternative hypotheses were: (1) a monophyletic LpOps6, LpOps7 and LpOps8, which are uniquely expressed in ME and (2) a monophyletic LpOps9, LpOps10, LpUVops1, LpUVOps2, LpArthOps1 and LpArthrop2, which all share a very similar intron/exon structure. To statistically test these hypotheses, we performed a Swofford–Olsen–Waddell–Hillis (SOWH) test (Swofford et al. 1996) for each scenario. We used the program SOWHAT (version 0.35) (Church et al. 2015) to carry out these analyses on an alignment consisting of the *Limulus* opsin proteins. We specified the PROTGAMMAWAG model as implemented in RAxML (version 8.1.21) (Stamatakis 2006) using the default 1000 replicates for both tests.

### Distributions of Opsin Transcripts and Proteins

#### RT-PCR

We used RT-PCR to probe for transcripts encoding each opsin in cDNAs from the following tissues: ME, VE, LE, brain, synganglion and pooled segmental ganglia from adult animals, and brain, tails and the synganglion pooled with all segmental ganglia from large juveniles. The primers we used in most screens (supplementary table S4, Supplementary Material online) were designed to amplify across an intron and eliminate the possibility of amplifying genomic DNA. The exceptions were screens for LpOps6 and 7, which lack introns. In all screens, we assayed cDNAs prepared from at least two different tissue collections and verified the identity of each PCR product by sequencing.

#### In Situ Hybridization

We prepared sense and antisense digoxigenin-labeled RNA probes from the full-length coding regions of LpOps1, LpOps5, LpUVOps1, and LpPerOps1 as described previously (Katti et al. 2010; Battelle et al. 2014, 2015). Because LpOps1, 2, 3 and 4 transcripts are nearly identical, the probe directed against LpOps1 will detect all four transcripts. We also prepared digoxigenin-labeled sense and antisense RNA probes from full-length clones of LpUVOps2 and LpArthops1, the 1008-bp fragment of LpOps10, and 585- and 592-bp fragments of LpC-Ops1 and LpC-Ops2, respectively. We used all probes at a final concentration of 1 µg/µl. We applied probes to whole mounts of ventral larval eyes dissected from adults and CNS tissues (brain, synganglion and segmental ganglia) dissected from large juveniles that had been fixed and processed for *in situ* hybridization as previously described (Jezzini et al. 2005) except that we exposed tissues to probes for 72 h at 65 °C and used the color development protocol described by (Seaver and Kaneshige 2006). The times for color development ranged from a few hours to 7 days. We photographed whole mounts with a Zeiss Discovery VS stereo microscope. Fixed, frozen sections of LE and ME were probed for opsin transcripts as described previously (Battelle et al. 2015).

#### Immunocytochemistry

We fixed, processed and cut serial frozen sections of CNS tissue from large juveniles as described previously (Katti et al. 2010). We sectioned brain, synganglia and segmental ganglia separately, and immunostained sections as detailed previously for myosin III (LpMyoIII), LpOps1–2 (Battelle et al. 2001); LpOps5 (Katti et al. 2010) and LpPerOps1 (Battelle et al. 2015). The specificity of each antibody was verified in the studies cited above. After immunostaining, we incubated some sections with DAPI to visualize DNA. We collected fluorescent images using a confocal microscope as described previously (Battelle et al. 2015).

## Results

### Genome Sequencing

We sequenced the genome of a single adult *Limulus* male using a combined approach of Roche 454 and Illumina sequencing. We used *K*-mer frequencies to estimate the genome size to be ∼1.5 gigabases (Gb). The assembly is comprised of 286,792 scaffolds with an N50 scaffold length of 238 kb and an N50 contig length of 11.4 kb (table 1). The assembled coverage is 18×, and the assembly spans 1.8 Gb. We removed all contaminating sequences from the assembly, trimmed vectors (X), and ambiguous bases (N). Additionally, shorter contigs (≤200 bp) were removed prior to public release. In table 1, we also compare our assembly of the *Limulus* genome with assemblies reported previously for *Limulus* and two other extant horseshoe crabs, *C. rotundicauda and T. tridentatus* (Nossa et al. 2014; Kenny et al. 2016).

**Table 1:** Statistics for the *Limulus polyphemus* Genome Assembly Described in This Study Compared with Those of Previous Genome Assemblies of *L. polyphemus, C. brotundicaudia* and *Tachypleus tridentatus*

	Current study	Nossa et al. (2014)	Kenny et al. (2016)			
Species	*L. polyphemus*	*L. polyphemus*		*L. polyphemus*	*C. rotundicauda*	*T. tridentatus*
Sequence length (bp)	1,828,256,766	1,229,280,963		1,446,611,838	1,577,921,537	1,532,106,426
Assembly gap length (bp)	122,485,139	456,658		NA	NA	NA
Scaffold number	286,792	896,522		NA	NA	NA
Scaffold N50 (bp)	254,089	2,929		NA	NA	NA
Contig number	469,509	6,614,434		4,214,715	2,312,916	772,557
Contig N50	11,441	418		466	1,356	586

Note.—bp, base pairs; NA, not applicable.

The annotation for *Limulus* was generated by the National Center for Biotechnology Information (NCBI). The analysis identified 22,129 genes and pseudogenes, 2066 transcripts, and a total of 23,287 coding DNA sequences. The mean length of all genes is 29,383 bp.

### Identification of *Limulus* Opsin Genes

We identified 18 *Limulus* opsin genes by BLASTing R-type and C-type opsin sequences against our *Limulus* genome assembly. The predicted proteins encoded by these genes are clearly opsins (supplementary fig. S1, Supplementary Material online). Each has seven predicted transmembrane domains, a predicted conserved lysine in helix VII that is critical for Schiff base binding of the chromophore, suggesting that each can form a photopigment, and acidic amino acids (glutamic acid/aspartic acid) at sites 83 and 181 (bovine rhodopsin numbering), which are potential sites for the Schiff base counter-ion in some R-type opsins (Porter et al. 2012). The sequences also have other motifs specific to different opsin classes (see below).

### Relationship of *Limulus* Opsins to One Another and to Opsins of Other Species

**Fig. 2.—:**
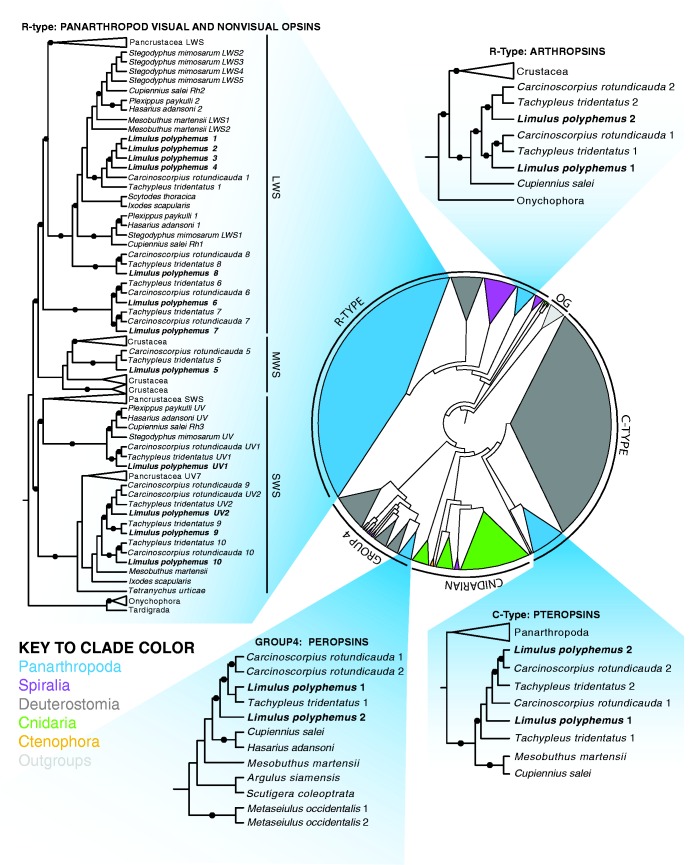
Opsin phylogeny. Maximum-likelihood tree of 743 genomic and expressed opsin sequences illustrating the four major evolutionary clades of opsins (C-type, R-type, Cnidarian, and Group 4), divided into subclades by major taxonomic groups (see key for color codes). Panarthropod groups are expanded to highlight the relationships among *Limulus polyphemus* (in bold) and arachnid and other xiphosuran opsin sequences. For the R-type clade containing the known panarthropod opsin genes, the spectral clades have been indicated as long wavelength sensitive (LWS), middle wavelength sensitive (MWS), and short wavelength sensitive (SWS). Black circles on branches indicate nodal support >80%.

To place the *Limulus* opsin genes within known opsin sequence diversity, we reconstructed a maximum-likelihood phylogeny that included 743 sequences representing known opsin and taxonomic diversity (fig. 2). Included in this tree are partial sequences of five opsins recovered from the scorpion genome (supplementary table S2, Supplementary Material online), only two of which (Mmops1 and Mmops2) were described previously (Cao et al. 2013). Mmops3 described by Cao et al. (2013) was not included because it lacks a lysine in the chromophore binding pocket and therefore is probably not an opsin. We also include partial sequences of 14 opsins recovered from the *T. tridentatus* genome and 15 from the *C. rotundicauda* genome (supplementary table S3, Supplementary Material online).

We placed *Limulus* opsins within three of the four major opsin groups: R-type, C-type and RGR/Go-type, also referred to as Group 4 opsins (Porter et al. 2012). Among the *Limulus* R-type opsin genes, we identified the following: seven long-wavelength sensitive (LWS) opsins (LpOps1–4, 6, 7 and 8), one middle-wavelength sensitive (MWS) opsin (LpOps5), one ultra-violet sensitive (UVS) opsin (LpUVOps1), three that are most closely related to pancrustacean UV7 opsins (LpOps9, 10 and UVOps2), and two arthropsins (LpArthOps1 and 2). Each of these opsins contains an eight-amino acid indel found in many arthropod opsins that lengthens cytoplasmic loop III (Porter et al. 2007). The sequence of this indel, which is highly conserved in crustacean R-type opsins, is also highly conserved in many *Limulus* R-type opsins (LpOps1–4, 5, 6, 8, 10 and UVOps1), moderately conserved in LpOps7, but poorly conserved in LpUVOps2, LpOps9 and the arthropsins (supplementary fig. S1, Supplementary Material online). The amino acid triplet characteristic of R-type opsins that activate the G-protein Gα_q/11_ (HPK/R) is conserved in all *Limulus* opsins within the R-type opsin group except for the two arthropsins (supplementary fig. S1, Supplementary Material online). This raises questions about the identity of the down-stream targets of arthropsins.

In addition to the R-type opsins, we identified two C-type opsins (LpCOps1 and 2) and two peropsins (LpPerOps1 and 2) in the *Limulus* genome. LpCOps1 has the amino-acid triplet NPQ, which is similar to and aligns with the sequence NKQ found in vertebrate C-type opsins thought critical for activating Gα_T_. The sequence of LpCOps2 in this region is not yet known. In LpPerOps1 and 2 the sequence at this site is NPR (supplementary fig. S1, Supplementary Material online), which is similar to and aligns with the sequence NPK in spider peropsins (Nagata et al. 2010; Eriksson et al. 2013) and the sequence HKK and NKK in mouse and human peropsins, respectively (Sun et al. 1997). The G-protein activated by peropsins, if any, is unknown.

In our analyses, most of the *Limulus* opsins form closely related clades with opsins from the other chelicerates: spider, tick, mite and scorpion. Furthermore, in the other two extant horseshoe crabs *T. tridentatus* and *C. rotundicauda*, we found the same diversity of opsins as we found in *Limulus*, including homologues of LpOps5, which cluster among crustacean MWS opsins (fig. 2). We assembled only one complete LpOps1-like sequence from the genomes of *T. tridentatus* and *C. rotundicauda*, but in the genomes of both of these species, we found nearly identical LpOps1-like sequences on multiple short contigs suggesting that they also have multiple Ops1-like genes.

**Fig. 3.—:**
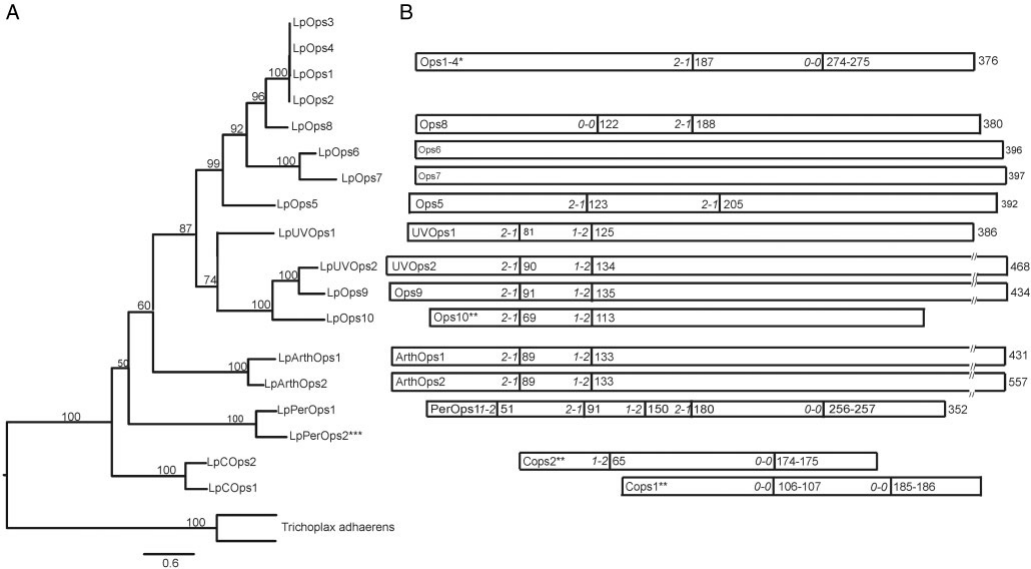
*Limulus* opsin gene structure. (*A*) Maximum-likelihood tree of *Limulus* opsin sequences determined from transcripts or from the genome (***). Outgroup opsins are from *Trichoplax adhaerens* (Accession numbers XP_002114592 and XP_002114593). Numbers on branches represent nodal support values calculated using rapid bootstrapping methods (Stamatakis et al. 2008; Pattengale et al. 2009; Stamatakis 2014). (*B*) Genomic structure of *Limulus* opsins. Rectangles represent the open reading frames of opsin transcripts that have been verified by sequencing. (**) Sequences that are not full length. Numbers at the right of the rectangles indicate the amino acid length of opsins for which the full-length sequence is known. The vertical lines within the rectangles show the positons of introns. Numbers on the right of each bar indicate the amino acid where the intron is located; the numbers in italics at the left indicate the phase of the intron. Intron locations and phases were deduced from the genomic assembly except for the introns in LpOps1–4 (*), which were determined by PCR and Sanger sequencing of genomic DNA (Dalal et al. 2003). Introns were identified from tBLASTn analyses of the *Limulus* genome. Phases were determined from translations of the relevant regions of the genome. The positions of opsin introns relative to one another were determined from a ClustalW alignment, which is shown in supplementary fig. S1, Supplementary Material online.

A number of *Limulus* opsin genes appear to be paralogous groups: LpOps1–4, LpOps6 and 7, LpUVOps2 and LpOps9, LpArthopsin1 and 2, LpPerOps1 and 2, and LpCOps1 and 2 (figs. 2 and 3*A*). Each set has an identical gene structure (intron location and phase) (fig. 3*B*) and encodes proteins that share 50% or greater sequence identity (supplementary fig. S2, Supplementary Material online). Most sets are encoded on different genomic scaffolds, but the genes encoding LpOps1–4 are on the same scaffold, and the proteins these genes encode are 99% identical to one another suggesting they are the result of tandem duplication events. LpOps1 and 2 transcripts can be distinguished unambiguously from one another by the sequences of their 3′-untranslated regions (Smith et al. 1993), but LpOps1, 3 and 4 genes can be distinguished from one another only by their intron sequences (Dalal et al. 2003).

It has recently been proposed that at least one whole-genome duplication event occurred in the stem ancestor of modern-day horseshoe crabs (Nossa et al. 2014; Kenny et al. 2016). This suggestion is based on the identity of pairs of loci with large numbers of shared paralogous gene pairs on different chromosomes including the presence of duplicated Hox and ParaHox clusters in *Limulus*, *T. tridentatus* and *C. rotundicauda.* To test if the paralagous opsin pairs on distinct scaffolds were consistent with the proposed ancient whole-genome duplication, we BLASTed these scaffolds against the human reference protein set and looked for other genes that might be shared between these scaffolds. We found examples of top BLAST hits on scaffolds that encode paralogous copies of the arthropsins, the peropsins and LpOps7 and LpOps6 and 8 (supplementary fig. S3, Supplementary Material online). LpOps6 and 8 are located in tandem on the same scaffold. These observations are consistent with the proposed whole-genome duplication. But LpOps5, 8, 10, and UVOps1 do not have paralogs. Thus, if a whole-genome duplication occurred, it must have been followed by significant opsin gene loss.

When we compared *Limulus* opsin genes with those we recovered from the genomes of two other extant horseshoe crabs, *T. tridentatus* and *C. rotundicauda*, we found the same complement of paralogous opsin pairs and nonparalogous opsins in all three species (fig. 2). This finding is consistent with the proposed whole-genome duplication occurring early in the xiphosuran linage (Kenny et al. 2016) and suggests that opsin gene loss also occurred early in the lineage. The argument for a whole-genome duplication event early in the xiphosuran lineage would have been strengthened had we discovered synteny between paralogous opsin pairs and other genes in the genome assemblies of *T. tridentatus* and *C. rotundicauda.* Unfortunately, we were unable perform these analyses because the genome assemblies of *T. tridentatus* and *C. rotundicauda* are too fragmented (table 1).

We examined gene structure to explore further relationships among *Limulus* opsins and between *Limulus* opsins and opsins from other species. We were particularly interested in the relationship between LpOps8 and the paralogs LpOps6 and 7. All three are uniquely expressed in MEs (see below) and are co-expressed in one population of ME photoreceptors (Battelle et al. 2015). Furthermore, LpOps6 and 7 are intronless paralogs, and as was mentioned above, the LpOps6 and 8 genes are located on the same scaffold within 5.5 kb of one another. While the linkage of LpOps6 and 8 is consistent with the occurrence of a recent tandem duplication event, our phylogenetic analyses (figs. 2 and 3*A*) suggest that LpOps8 is more closely related to LpOps1–4 than it is to LpOps6 and 7. To be sure that LpOps6, 7, and 8 are not a monophyletic clade, we ran a Swofford–Olsen–Waddell–Hillis (SOWH) test, which rejected this alternative hypothesis (*p*≤0.001). Our analysis of gene structure, which shows that intron two in LpOps8 aligns with intron one of LpOps1–4, provides additional support for a close relationship between LpOps1–4 and 8 (figs. 2 and 3*B*).

**Fig. 4.—:**
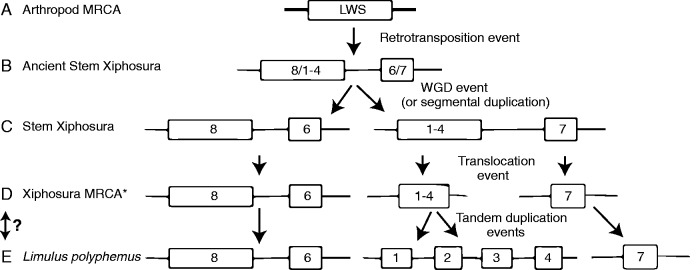
Evolution of LWS opsins in the *Limulus* lineage. There are several ways to reconstruct the events that led to the current number and arrangement of LWS opsin genes in *Limulus*, but this figure represents the most likely hypothesis based on the tree in fig. 2 and parsimony principles. (*A*) There was a single LWS gene in the ancestor of all arthropods. (*B*) A retrotransposition event occurred after Xiphosura diverged from the rest of Arthropoda leading to the ancestor of LpOps6 and LpOps7; both of these opsins lack introns. (*C*) A whole-genome duplication (WGD) or segmental duplication event in the stem of Xiphosura led to four LWS opsins. This is supported by the presence of similar protein kinases (both match best to human PRKX) on the scaffold that contains LpOps6 and 8 as well as the scaffold that contains LpOps7. (*D*) LpOps1–4 and LpOps7 are on separate scaffolds in the *Limulus* genome suggesting that a translocation event occurred prior to the most recent common ancestor (MRCA) of Xiphosura. (*E*) There are four highly similar tandem copies in the *Limulus polyphemus* genome likely the result of recent tandem duplication events. The swapping arrows and question mark indicate that it is not possible to definitively determine the order of (*D*) and (*E*) given the poor resolution for the other Xiphosura genomes; therefore, it is unclear if there are four LpOps1–4 genes in those assemblies, or single genes that descended from the ancestral LpOps1/4. It is also possible that the LpOps1/4 gene(s) could be linked to LpOps7 in these other horseshoe crab genomes.

Using our phylogeny, we reconstructed an evolutionary scenario explaining the origins of the *Limulus* LWS genes (fig. 4). The scenario is consistent with an ancient whole-genome duplication event occurring in the Xiphosura stem lineage. Furthermore, the scenario suggests that the synteny between the median-eye specific LpOps6 and 8 opsins has been maintained for a very long time and therefore may have a strong functional significance.

We found that opsins from different clades have the same gene structure. For example introns 4 and 5 in LpPerOps1, a RGR/Go-type opsin, match in position and phase the introns in LWS LpOps1–4. We also found that opsins LpUVOps1 and 2, LpOps9 and 10, and LpArthops1 and 2 each has two introns with identical positions and phases. Our maximum-likelihood trees (figs. 2 and 3*A*) place these opsins in three distinct R-type opsin clades: (1) chelicerate UVS opsins within the larger pancrustacean short-wavelength sensitive (SWS) opsin clade, (2) chelicerate UVOps2, Ops9 and Ops10 within the pancrustacean UV7 opsin clade, and (3) the more distantly related arthropsins, containing both crustacean and chelicerate sequences. Their identical gene structure suggested they might be a monophyletic group, but based on the results of a SOWH test, we rejected this alternative hypothesis in favor of the relationship shown in the maximum-likelihood tree (*p*≤0.001).

Our observation that different clades of *Limulus* R-type opsins have the same gene structure prompted us to examine whether opsins from other species also have this structure. To test this, we used TBLASTN to identify R-type opsin genes in selected genomes using LpOps10 as query. We recovered R-type opsin genes with the same two introns from representative species of arthropods, annelids, echinoderms and mammals (supplementary fig. S4, Supplementary Material online). We found that the arthropsins of the water flea *Daphnia pulex* also have the same two introns (not shown). By contrast, the intron structure of the LWS *Limulus* opsins, which are most closely related to the SWS *Limulus* opsins based on sequence homology and phylogenetic analyses, do not share these two introns.

We found that intron-exon junctions in other *Limulus* opsins are conserved in distantly related lineages. The three introns in *Limulus* C-type opsins are strictly conserved with introns 1, 3 and 4 in bovine rhodopsin (supplementary fig. S5, Supplementary Material online), although the donor sequence of the first intron of LpCops2 is AC instead of the canonical GT (Burset et al. 2000) identified in bovine rhodopsin (Nathans and Hogness 1983) and in all other introns of *Limulus* opsins (this study, not shown). Introns 1, 3 and 5 of LpPerOps1 are strictly conserved with peropsin genes of vertebrates, hemichordates and several other invertebrates (Albalat 2012). Intron 1 in LWS LpOps1–4 and 8 is strictly conserved in insect LWS opsins and the second intron in LWS LpOps1–4 matches in position and phase an intron in some insect LWS opsins (supplementary fig. S6, Supplementary Material online). Although the introns of LpOps5 are unique among *Limulus* opsins (fig. 3*B*, supplementary fig. S1, Supplementary Material online), intron 2 of LpOps5 matches the position and phase of intron 4 in each of the *D. pulex* opsins most closely related to LpOps5 within the larger MWS clade (fig. 2; supplementary fig. S7, Supplementary Material online) but does not align with any introns in *D. pulex* opsins in the second subgroup within the MWS clade (not shown).

### Opsin Expression

#### Organization of the Limulus Visual and Nervous Systems

The schematics of the *Limulus* visual and central nervous systems in fig. 1 will orient readers to structures we assayed for opsin expression. In fig. 1*A*, we show the locations of the three types of *Limulus* eyes, and in the central cut-away, we show the location the brain. Also, in the central cut-away, we show the optic nerves of the VEs, which in adult animals, project anteriorly from the brain and terminate in a pair of end organs attached to a specialized region on the ventral cuticle. Each end organ typically contains a large cluster of photoreceptor cell bodies. In newly hatched animals and juveniles, ventral photoreceptor cell bodies lie close to the anterior brain, and even in adults, some ventral photoreceptor cell bodies remain on the brain (Chamberlain and Wyse 1986). On the brain’s dorsal side (fig. 1*B*), central projections from the lateral, median and ventral optic nerves can be seen, as well as the lamina (first optic ganglia), medulla (second optic ganglia) and central body. The corpora pedunculata, also called mushroom bodies, are on the ventral side of the brain as are most neuronal cell bodies of the synganglion and segmental ganglia (fig. 1*C*).

#### Tissue Distribution Assayed with RT-PCR

We screened for transcripts encoded by each of the 18 opsin genes in cDNA prepared from adult eyes and in a cDNA library prepared from young juveniles. Except for LpPerOps2, we found that each of the opsin genes identified in the genome is expressed in one or more of these tissues, with one qualification. Because transcripts encoded by the LpOps1, 3, and 4 genes are nearly identical (Dalal et al. 2003), we have no direct evidence that all three genes are expressed.

**Fig. 5.—:**
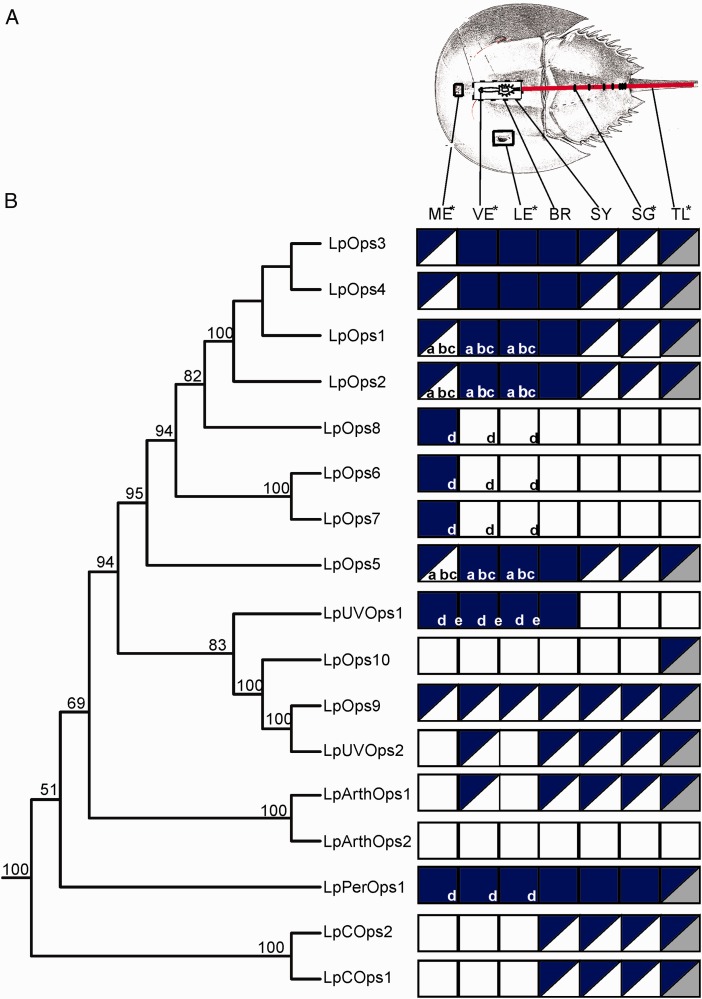
Distribution of opsin transcripts and protein in *Limulus* eyes and nervous system. (*A*) Schematic of *Limulus* showing the locations of tissues tested for opsin transcripts by PCR and *in situ* hybridization and for opsin protein by immunocytochemistry. (*B*) (Left) Phylogenetic tree of *Limulus* opsins is modified from that shown in fig. 3. LpPerOps2 is not included here because transcripts encoding this opsin were not detected in any tissues assayed. LpArthOps2 is included because its transcripts were detected in the CNS of young juveniles. (*B*) (Right) Tissue distribution of the opsin shown at left. (Top) Tissues assayed. Tissues in which photosensitive cells have been detected by electrophysiology are indicated with an asterisk (*). (See Discussion for details). Solid blue box, transcript detected by PCR and *in situ* hybridization/immunocytochemistry. Blue-white box, transcript detected by PCR but not by *in situ* hybridization/immunocytochemistry. Blue-gray box, transcript detected by PCR and not tested by *in situ* hybridization/immunocytochemistry. Solid white box, transcript not detected by PCR and therefore not tested with *in situ* hybridization. The distribution of some opsins in eyes was determined previously. (*a*) Smith et al. 1993, (*b*) Dalal et al. 2003, (*c*) Katti et al. 2010, (*d*) Battelle et al. 2015, and (*e*) Battelle et al. 2014. ME, median eye; VE, ventral eye; LE, lateral eye; BR, brain; SY, synganglion; SG, segmental ganglia; TL, tail.

Nine *Limulus* opsins and their expression patterns in eyes were described in detail previously: LpOps1 and 2 (Smith et al. 1993; Dalal et al. 2003), LpOps5, (Katti et al. 2010), LpOps6, 7, 8 and LpPerOps1 and 2 (Battelle et al. 2015), and LpUVOps1 (Battelle et al. 2014). In the current study, we screened for transcripts encoded by the newly identified opsin genes (LpUVOps2, LpOps9 and 10; LpArthops1 and 2; and LpCOps1 and 2) in cDNA from adult eyes, the CNS of adults and the CNS and tail of older juveniles. We also searched for transcripts encoding the previously identified opsins in cDNA from the CNS of adults and the CNS and tail of older juveniles. Because all larval eyes contain the same two classes of photoreceptors (Harzsch et al. 2006; Battelle et al. 2014) we consider results from assays of adult VEs as representative of all three types of larval eyes. Figure 5 summarizes results obtained from adult eyes and CNS and from juvenile tails. The distribution of opsin transcripts in the CNS of the older juveniles, which were also used in the *in situ* assays, was the same as that observed in adults. Supplementary Figure S8, Supplementary Material online shows sample results from the PCR reactions used to generate the results in fig. 5.

We detected transcripts of all opsins in one or more of the eyes, CNS or tail, except for LpPerOps2, which is not expressed in any of the tissues we assayed. LpArthops2 transcripts were not detected in any tissues we assayed from older juveniles or adults, but we obtained a full length clone of this opsin from the CNS of young juveniles indicating it may be expressed early in development. We detected most opsin transcripts in multiple tissues. Exceptions were transcripts encoding LpOps6, 7 and 8, which we detected only in MEs, and LpOps10, which we detected only in the tail. We found that three of the sets of opsin paralogs have the same expression pattern (i.e., LpOps1–4; LpOps 6 and 7; LpCOps1 and 2) and three do not (i.e., LpOps9 and LpUVOps2; LpPerOps1 and 2; LpArthop1 and 2). We detected LpPerOps1 in all tissues assayed but LpPerOps2 in none. Similarly, we detected LpArthop1 throughout the CNS, in the tail and VEs, but we found LpArthop2 in none of these tissues in older juvenile or adult animals.

#### Cellular Distribution Assayed with In Situ Hybridization and Immunocytochemistry

We used *in situ* hybridization assays to examine the cellular distribution of opsin transcripts in adult eyes and in the CNS of older juveniles. We assayed for opsin proteins in these same tissues using immunocytochemistry and specific antibodies directed against LpOps1–4, 5, 6, UVOps1 and PerOps1. We were unable to apply these techniques to the tail because of the unique challenges of doing morphology on the tail.

#### Eyes

As was described above, our PCR screens detected LpOps9 transcripts in each eye type and LpUVOps2 and LpArthOps1 transcripts in VEs (fig. 5). However, our *in situ* hybridization assays for these transcripts in eye tissues consistently produced negative results. By contrast, using identical *in situ* hybridization protocols, we detected LpOps1, 5 and LpUVOps1 in LEs, LpOps 6, 7, 8 and LpUVOps1 in MEs and LpOps1, 5 and LpUVOps1 in VEs (Battelle et al. 2014, 2015).

#### CNS

**Fig. 6.—:**
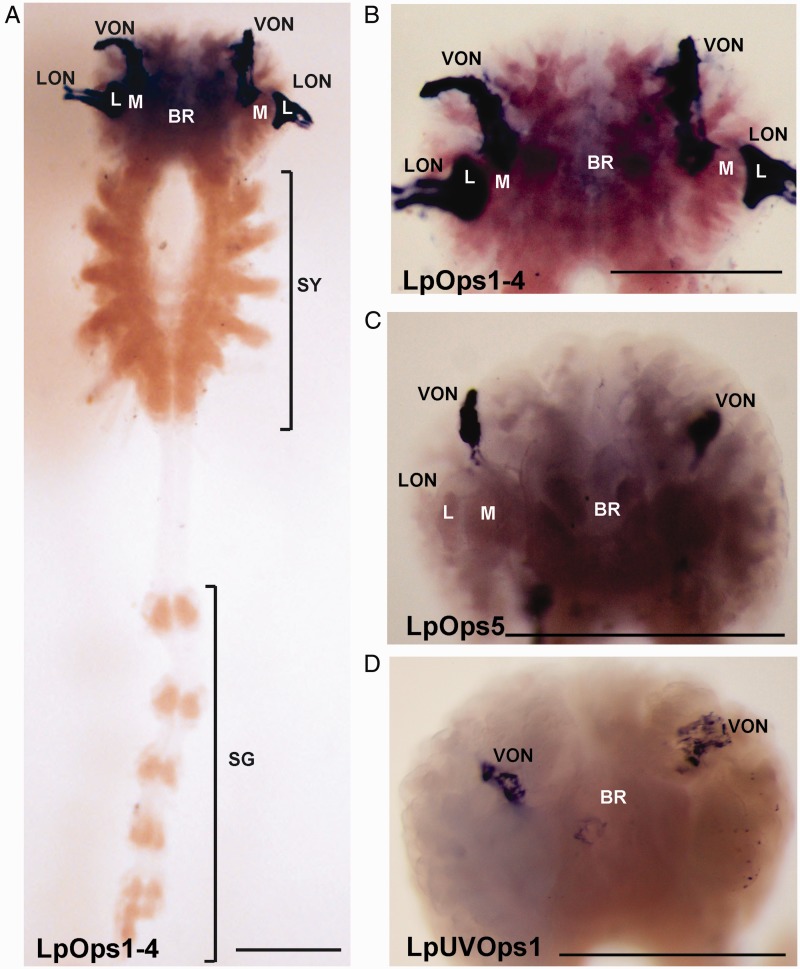
LpOps1–4, 5 and UVOps1 transcripts detected by *in situ* hybridization. LpOps1–4, 5 and UVOps1 transcripts were detected in the brain in processes from lateral eyes and cell bodies and processes from ventral eyes, but not elsewhere in the central nervous system. (*A*) CNS whole-mount from a large juvenile *Limulus* incubated with an antisense probe targeting LpOps1–4 transcripts. A dorsal view is shown. LpOps1–4 transcripts were consistently detected in lateral optic nerves (LON) as they enter the brain (BR) and in the lamina or first optic ganglia (L) where axons from the large retinular cells of the lateral compound eye terminate. Cell bodies and processes of ventral optic nerves (VON) that project to the medulla or second optic ganglia (M) were also labeled, but no transcripts were detected in the synganglion (SY) or segmental ganglia (SG). (*B*) Enlarged view of the dorsal brain (BR) shown in (*A*), (*B*), and (*C*). Dorsal view of the brain of an older juvenile incubated with antisense probe targeting LpOps5 and LpUVOps1 transcripts, respectively. Only cell bodies and processes of the ventral photoreceptors were labeled. Scale bars, 1 mm.

We consistently detected LpOps1–4 transcripts in processes of LE photoreceptors as they enter the brain and terminate in the lamina, in VE photoreceptor cell bodies located close to the brain and in VE processes that project to and terminate in the medulla (fig. 6*A* and *B*). We also detected LpOps5 and LpUVOps1 transcripts in VE photoreceptor cell bodies and their processes (fig. 6*C* and *D*). We did not detect LpOps1–4, 5 or LpUVOps1 transcripts in cell bodies or processes elsewhere in the brain, and although our PCR screen revealed LpOps1–4 and 5 transcripts in the synganglion and abdominal ganglia (fig. 5), we were unable to detect transcripts in these tissues with *in situ* hybridization assays even after 7 days of development. Using antibodies that detect LpOps1–4, 5, and UVOps1 proteins in rhabdoms of photoreceptors in eyes (Battelle et al. 2001, 2014; Katti et al. 2010), we detected these opsins in ventral photoreceptor cell bodies on the brain, but not in LE or VE processes or elsewhere in the brain (not shown). We also were unable to detect these opsins in sections of the synganglion or segmental ganglia. Our *in situ* hybridization assays for LpUVOps2, LpOps9, LpArthops1 and LpCOps1 and 2 transcripts in whole mounts of brain and ventral nerve cord of older juveniles also produced negative results.

**Fig. 7.—:**
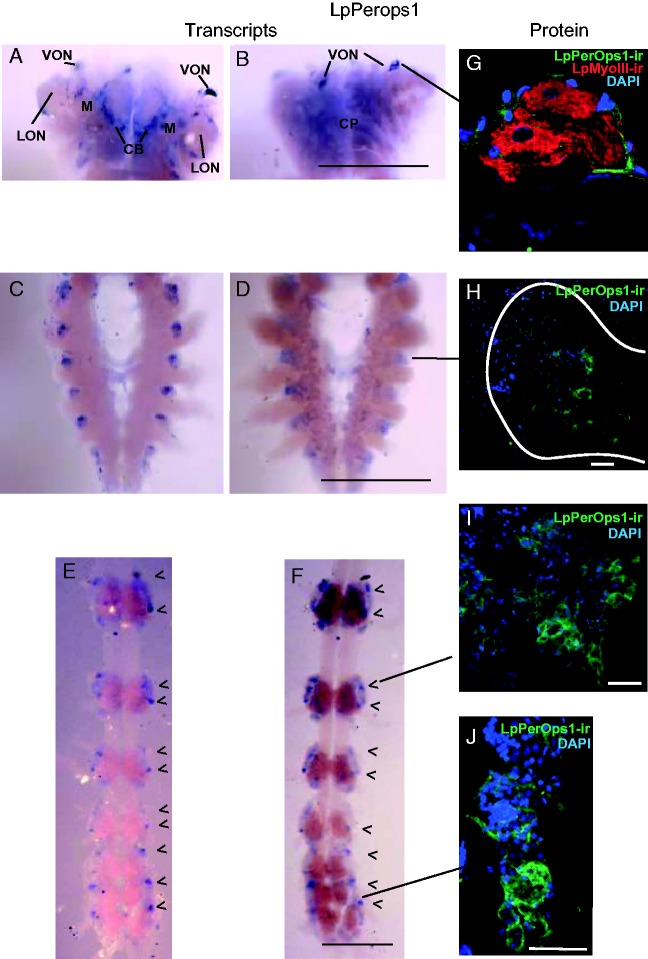
Distribution of LpPerOps1 transcripts and protein. LpPerOps1 transcripts and proteins were detected in the brain, synganglion and segmental ganglia of older juveniles. (*A–F*) Representative whole-mount of the CNS from an older juvenile incubated with an antisense probe targeting LpPerOps1 transcripts. Dorsal views (*A*, *C*, and *E*) and ventral views (*B*, *D*, and *F*) of the brain (*A* and *B*), synganglion (*C* and *D*) and segmental ganglia (*E* and *F*). In the brain (*A* and *B*), LpPerOps1 transcripts were detected in the ventral optic nerve (VON), at the periphery of the lateral optic nerve (LON), in fibers that may be in the central body (CB), which is visible on the dorsal side of the brain. The locations of the second optic ganglia or medulla (M) are indicated. Transcripts were not detected in the corpora pedunculata (CP) on the brain’s ventral side. Transcripts in the synganglion (*C* and *D*) were associated with cell clusters located between the large nerve roots projecting to the periphery. In segmental ganglia (*E* and *F*), transcripts were typically associated with two bilateral cell clusters in each ganglion (arrow heads). Scale bars, 1 mm. (*G*–*J*) Fixed, frozen sections of cell clusters from different regions of the juvenile CNS that were immunostained for LpPerOps1 (Green) and incubated with DAPI to reveal nuclei (Blue). (*G*) A small cluster of giant ventral photoreceptors on the brain. Ventral photoreceptor cell bodies, identified with LpMyoIII-immunoreactivity (red) (LpMyoIII-ir), a marker for photoreceptors in each of the eyes (Battelle et al. 2001), are surrounded by LpPerOps1-immunoreactive glia, as was described previously (Battelle et al. 2015). (*H*) Immunostained cell clusters from the synganglion. The periphery of the cluster is outlined. (*I* and *J*) Cell clusters from different segmental ganglia, as indicated. In each cell cluster examined, LpPerOps1-immunoreactivity (LpPerOps1-ir) surrounded neurons and was not uniform throughout the clusters. Scale bar, 50 µm.

By contrast, our *in situ* hybridization assays revealed LpPerOps1 transcripts throughout the CNS (fig. 7*A–F*). In the brain, the antisense probe targeting LpPerOps1 labeled ventral optic nerves most intensely (fig. 6*A* and *B*), which is consistent with our finding of LpPerOps1 immunoreactivity in glia surrounding ventral photoreceptors (fig. 7*G* and Battelle et al. 2015). We detected no LpPerOps1 transcripts in the corpora pedunculata (fig. 7*B*), but transcripts were consistently detected in cells at the periphery of the lateral optic nerves and in what appear to be fibers within the central body (fig. 7*A*). In the synganglion, transcript was associated with neuronal clusters located between large nerve bundles projecting to the periphery. These cell clusters were most evident when viewed from the dorsal side (fig. 6*C*). In segmental ganglia, transcript was consistently associated with two or three bilateral neuronal clusters in each ganglion. When the brain, synganglion and segmental ganglia were immunostained for LpPerOps1 protein, we detected LpPerOps1 immunoreactivity surrounding neurons indicating LpPerOps1 protein is present in these regions (fig. 7*G–J*).

## Discussion

In this study, we sequenced and assembled the genome of the American horseshoe crab *L. polyphemus*. We used these data to identify and phylogenetically classify 18 opsin genes in this genome into three of the four currently recognized major opsin groups and gain insights into a number of key events in arthropod opsin evolution. We further showed that gene structure supports the placement of the opsins within these major clades and provides new insights into the relationship of the major clades to one another. In our studies of opsin expression, we detected transcripts for all of these opsins by RT-PCR in the eyes, CNS or tail of adults or older juveniles except for LpArthops2, which was detected only in the CNS of young juveniles and LpPerOps2, which was not detected in any tissues we assayed. We also showed that LpPerOps1 is expressed in glia throughout the CNS. We were unable to identify opsin-expressing neurons in the CNS by *in situ* hybridization or immunocytochemistry suggesting that opsin transcripts levels are low (or absent) in neurons, which raises questions about their functional significance. However, as is discussed below, electrophysiological studies have shown that photosensitive cells are present in *Limulus* CNS and tail. Our results provide a list of opsin candidates that may contribute to this extraocular photosensitivity.

### Arthropod Opsin Evolution

Our data point to key events in the evolution of arthropod opsins. The large number of opsins in horseshoe crabs—18 in *Limulus*, and at least 14 and 15 in *T. tridentatus* and *C. routundicauda*, respectively—compared with the number so far identified in other chelicerates—six in transcriptomes from the jumping spider *C. salei* (Eriksson et al. 2013), six in the spider *S. mimosarum*, and five that we recovered from the genome of the scorpion *M. martensii*)—is consistent with a whole-genome duplication event early in the xiphosuran lineage (Nossa et al. 2014; Kenny et al. 2016). Similar patterns are not present in scorpions or spiders, which suggests that the event occurred after Xiphosura diverged from the rest of Euchelicerata. As the same complement of paralogous opsin pairs and nonparalogous opsins are present in all three horseshoe crab species examined, we suggest that significant opsin gene loss also occurred in the stem of the xiphosuran linage.

Several opsins appear to have evolved only in xiphosurans. For example, LpOps6, 7, and 8, which are ME specific in *Limulus*, appear to have radiated from an ancestral LWS opsin after Xiphosura diverged from the rest of Euchelicerata. We also identified opsins in xiphosurans that appear to have been lost in other euchelicerates. We found LpOps5 homologs in each of the extant horseshoe crabs examined, and homologues are present in crustaceans. Therefore, an LpOps5 homologue was likely present in the last common ancestor of crustaceans and euchelicerates, but was lost in the lineage leading to scorpion and spiders.

Long-wavelength sensitive opsin genes have expanded in *Limulus* by apparent tandem gene duplications to form the LpOps1–4 cluster. This expansion may be of particular functional significance for *Limulus* vision**.** LpOps1–4 is co-expressed with LpOps5 in rhabdomes of *Limulus* LE retinular cells, but the proteins encoded by the LpOps1–4 genes are ∼4 times more abundant than those encoded by LpOps5 (Katti et al. 2010). Furthermore, while the LpOps5 protein concentration in rhabdomes is relatively stable day-to-night, probably because of a steady rate of turnover, the LpOps1–4 protein concentration in rhabdomes changes dramatically. It falls to 50% of its nighttime peak early in the day in response to the onset of light, and it is restored to its nighttime peak concentration by 4 h after sunset in response to darkness and signals from an internal circadian clock (Battelle 2013; Battelle et al. 2013). In addition, LpOps1–4-containing rhabdomeric membranes are actively shed and renewed throughout the day (Sacunas et al. 2002; Katti et al. 2010; Battelle et al. 2013). This indicates that processes involved in LpOps1–4 protein turnover are particularly active and highly regulated. They are thought to contribute to a dramatic nighttime increase in the sensitivity of the LE to light (Barlow et al. 1977), and thus the animal’s ability to find mates while spawning at night (Barlow et al. 1982).

The eyes of some species of scorpions and spiders also show dramatic changes in sensitivity that are controlled in part by a circadian system similar to that in *Limulus* (Fleissner and Heinrichs 1982; Fleissner 1983; Fleissner and Fleissner 1988; Yamashita 2002). These are correlated with daily changes in rhabdome structure (Fleissner and Fleissner 1988; Grusch et al. 1997), but in scorpions and spiders it is not yet known whether changes in sensitivity and structure correlate with changes in opsin protein concentrations in rhabdomeric membranes. It is interesting to note, however, that, like *Limulus*, the nocturnal spider *S. mimosarum* (Crouch and Lubin 2000) has an expanded repertoire of LWS opsin (fig. 2).

### Insights from Gene Structure

The structure of *Limulus* opsin genes confirms their placement in phylogenetic trees based on sequence homology and provides new insights into the relationships among opsins. Gene structure is classically considered highly conserved in evolution and therefore a feature that can provide insights into relationships among gene families (Rokas and Holland 2000). The present study and that of Dalal et al., (2003) are the first to describe genomic structures for chelicerate opsins.

Our finding that the introns of LpCOps1 and 2 are strictly conserved with introns 1, 3 and 4 of all known vertebrate C-type opsins and with introns in C-type opsin genes of other invertebrates including insects (e.g., *Tribolium*), and crustaceans (e.g., *D. pluex*,) (Fridmanis et al. 2007), supports their placement within the large C-type opsin group and extends the idea of the homology of all bilaterian C-type opsins. Similarly, our placement of LpPerOps1 among the peropsins within the RGR/Go group is supported by our finding that introns 1, 3 and 5 of the LpPerOps1 gene are identical to those in peropsin genes of vertebrates, hemicordates and several other invertebrates (Albalat 2012). The conserved introns we identified in *Limulus* SWS opsins and arthropsins (fig. 2) and recovered in R-type opsins from diverse species (supplementary fig. S4, Supplementary Material online), are consistent with the placement of these opsins within the R-type opsin group and suggest the structure of *Limulus* SWS and arthropsin genes is ancient among R-type opsins.

Surprisingly, *Limulus* LWS opsin genes, which are closely related to the SWS opsins based on sequence homology and phylogenetic placement, have a very different structure (fig. 3). However, the structure of *Limulu*s LWS R-type opsin genes matches that of LWS R-type opsin genes in insects (supplementary fig. S6, Supplementary Material online). This finding supports placement of LpOps1–4 and 8 among other LWS R-type opsins and suggests the structure of LpOps1–4 is ancient among arthropod LWS R-type opsins.

Our analyses of gene structure points further to a relationship between LWS LpOps1–4 and LpPerOps1. The introns in LWS LpOps1–4 align with and match the phase of introns 4 and 5 in LpPerOps1. As was described above, the introns of LpOps1–4 are probably deeply rooted in the phylogeny of LWS arthropod opsins, and intron 5 in LpPerOps1 is highly conserved in all peropsins. The apparent relationship between LWS opsins and peropsins revealed by gene structure was unexpected based on the phylogeny shown in fig. 2, although this relationship has been suggested in previous studies (Porter et al. 2012). Together these data show remarkable conservation of several intron-exon boundaries, which both support our phylogenetic classifications, and also suggest higher order relationships between major classes of opsins. In addition, the high level of intron conservation suggests a highly conserved regulatory role of these introns (e.g., mRNA stability, nuclear transport, etc.).

Curiously, LpOps5 was the only chelicerate opsin in a clade originally considered unique to crustaceans (Kashiyama et al. 2009). In the present study, we added to this MWS clade two LpOps5 homologues from other extant horseshoe crab species; thus strengthening the idea that an LpOps5 homolog was present in the last common ancestor of arthropods (Henze and Oakley 2015). This idea is further supported by our observation that the LpOps5 gene has an intron in common with some *Daphnia* MWS opsins (supplementary fig. S7, Supplementary Material online).

### Expression Pattern of the Full Repertoire of *Limulus* Opsins

#### Eyes

In previous studies, we showed by RT-PCR and immunocytochemistry or *in situ* hybridization that ten different opsins are expressed in eyes. The RT-PCR screens in the present study added LpOps9 to the list of opsin transcripts detected in each eye type and LpUVOps2 and LpArthops1 to the list in larval eyes. However, we were unable to verify their expression by *in situ* hybridization. These findings are reminiscent of results from our previous expression studies of LpOps1–4 and 5 in MEs. In MEs, we routinely detected LpOps1–4 and 5 transcripts by RT-PCR (Smith et al. 1993; Katti et al. 2010; Battelle et al. 2015) but not by *in situ* hybridization (Katti et al. 2010; Battelle et al. 2015). We were also unable to detect LpOps1–4 or 5 proteins in MEs by immunocytochemistry using antibodies that routinely detect them in photoreceptors of LEs and larval eyes (Katti et al. 2010; Battelle et al. 2015). Thus, the functional significance of opsin transcripts detected by RT-PCR only must be viewed with caution, especially in tissues where other opsins are clearly expressed.

#### CNS

We anticipated finding LpOps1–4, and 5, LpUVOps1 and LpPerOps1 transcripts in the brain because all are expressed in VE photoreceptor cell bodies, and these are often located on the anterior brain (fig. 6 and Chamberlain and Wyse 1986). Indeed, when we assayed for opsin transcripts in cDNA prepared from brains from which most VE photoreceptor cell bodies had been removed by cutting away a portion of the anterior brain, we did not detect LpUVOps1. This suggests VE photoreceptor cell bodies are the source of LpUVOps1 transcripts in the brain, and as we did not detect LpUVOps1 transcripts elsewhere in the CNS or tail, we conclude that LpUVOps1 is eye-specific. By contrast LpOps1–4, 5 and LpPerOps1 transcripts were detected in cDNA from brains lacking most ventral photoreceptor cell bodies (supplementary fig. S8, Supplementary Material online) suggesting these transcripts are also present elsewhere in the brain.

Our *in situ* hybridization assays revealed that major sources of LpOps1–4 transcripts in the brain are the axons of lateral and VE photoreceptors (figs. 1*B*, 6*A* and *B*). The *in situ* labeling of LpOps1–4 transcripts in these axons was so intense that it resembled labeling seen in tract-tracing studies of lateral and ventral optic nerves (Chamberlain and Barlow 1980; Calman and Battelle 1991; Calman et al. 1991) revealing photoreceptor axon terminals in optic ganglia. The intense labeling of LpOps1–4 transcripts was particularly surprising in lateral optic nerves because in the older juveniles we used in our *in situ* hybridization assays, the lateral optic nerve is ∼15 mm long. Active transport of transcripts long distances to axon terminals is commonly observed for transcripts encoding proteins that are translated and present in axons and terminals (Giuditta et al. 2008). However, this mechanism does not seem relevant to LpOps1–4 transcripts, because LpOps1–4 proteins are detected only in the specialized rhabdomeric membranes in photoreceptor cell bodies. The presence of LpOps1–4 transcripts in lateral and ventral optic nerves may reflect a particularly high level of these transcripts in photoreceptors, perhaps a consequence of transcription from all four genes in the LpOps1–4 gene cluster. High LpOps1–4 transcript levels are also consistent with the finding mentioned above that LpOps1–4 proteins are ∼4 times more abundant in LE photoreceptors than LpOps5 (Katti et al. 2010), which is encoded by a single gene.

Lateral eyes and VE axons may also be the sources of LpOps5, 9, 10, LpUVOps2 and LpArthops1 transcripts in brain as each is present in eye photoreceptors (fig. 5). Our inability to detect them in optic nerves by *in situ* hybridization suggests their levels in axons are low. ME photoreceptors expressing LpOps6, 7, and 8 also project to the brain (fig. 1*B*), but these transcripts are not detected in brain even by RT-PCR. If LpOps6, 7 and 8 transcripts are present in median optic nerves where they enter the brain, their levels must be extremely low. This may be because only ∼30% of ME photoreceptors express these opsins (Nolte and Brown 1972; Battelle et al. 2015).

Axons from photoreceptors in eyes cannot be the only source of opsin transcripts detected by RT-PCR in the brain and elsewhere in the CNS and tail. For example, we detected C-type opsin transcripts in the brain, and they are not expressed in any of the eyes. Furthermore, the opsins we detected in the synganglion, segmental ganglia and tail cannot be explained by input from eyes. A clear concern is that, except for LpPerOps1, which is discussed separately below, we were unable to identify any opsin expressing cells in the CNS by *in situ* hybridization. This raises questions about their functional relevance. On the other hand, some of the opsins we detected in the CNS and tail, even though expressed at low levels, may contribute to the extraocular photosensitivity that has been described in *Limulus.*

#### Extraocular Photosensitivity

No photosensitive cells have been identified in the brain, except for ventral larval eye photoreceptors located at the brain, and no photosensitive cells have been identified in the synganglion. This may be because there has been no systematic search for photosensitive cells in these tissues. Photosensitive cells have been described in segmental ganglia (Mori et al. 2004), and there is good evidence that the tail is photosensitive (Hanna et al. 1988; Renninger et al. 1997). The question most relevant to the current study is: Which of the opsins expressed in these tissues might contribute to this extraocular photosensitivity?

Photosensitive cells in segmental ganglia were identified with intracellular recordings which showed that each ganglion contains one or several photoreceptors, and that all photoreceptors so far examined are maximally sensitive to light at 425nm (Mori et al. 2004). This suggests that LpOps1–4 and 5, with maximum sensitivities at ∼520 nm, and LpUVOps2 are not involved. The remaining candidates are the C-type opsins, LpOps9 and LpArthops1. Photosensitivity in the tail was demonstrated by showing that the phase of the animal’s circadian clock can be shifted by illuminating the tail with broad spectrum light (Hanna et al. 1988). As the spectral sensitivity of the response was not investigated further, all of the opsins we detected in the tail are potentially involved. However, LpOps10 is of particular interest because its transcripts are tail-specific.

#### Peropsin

Peropsin is expressed in eyes and throughout the CNS. The results described here provide the first clear example of peropsin expression outside of eyes. Peropsin proteins were previously identified in the eyes of mammals (Sun et al. 1997) and other vertebrates (e.g., Bailey and Cassone 2004), in some but not all spider eyes (Nagata et al. 2010; Eriksson et al. 2013) and in *Limulus* eyes (Battelle et al. 2015). Peropsin transcripts have been detected in spider brain (Eriksson et al. 2013) and in transcriptomes of crustaceans, myriapods and insects (Henze and Oakley 2015), but the cellular distributions of these transcripts are not known. Where peropsin distribution has been examined, it is consistently found in glia or pigment cells most often, but not exclusively, surrounding photoreceptors. Based on its frequent association with photoreceptors, peropsins have been postulated to play a role in vision, possibly as a photoisomerase (Sun et al. 1997; Chen et al. 2001; Nagata et al. 2010).

Peropsins may function in vision, but the broad distribution of LpPerOps1 in the CNS we observe suggests it has other functions as well. Because the distribution of LpPerOps1 is not uniform in the CNS, we considered it might be specifically associated with opsin-expressing cells. But we think this unlikely. Nothing is known about the distribution of photosensitivity in the synganglion, but it seems unlikely that all the cells surrounded by LpPerOps1-expressing glia in this ganglion are photoreceptors. The number of cells surrounded by LpPerOps1-expressing glia in segmental ganglia also seems much larger than the 2% of cells thought to be photosensitive (Mori et al. 2004). Our findings add to the puzzle of peropsin function. Clearly, many more studies are required to clarify its role in eyes and in the CNS.

## Conclusions

These analyses of the full repertoire of opsins from the *Limulus* genome provide unprecedented insight into the visual system of a chelicerate. As such, these data are key to reconstructing the most recent common ancestor of arthropods and providing fundamental evolutionary insights into processes that shaped the immense diversity of visual systems found in Arthropoda.

## Supplementary Material

Supplementary tables S1–S4 and figs. S1–S8 are available at *Genome Biology and Evolution* online (http://www.gbe.oxfordjournals.org/).

Supplementary Data

## References

[evw100-B1] Albalat R. 2012 Evolution of the genetic machinery of the visual cycle: a novelty of the vertebrate eye? Mol Biol Evol. 29:1461–1469. 22319134 10.1093/molbev/msr313

[evw100-B2] Bailey MJ Cassone VM. 2004 Opsin photoisomerases in the chick retina and pineal gland: characterization, localization, and circadian regulation. Invest Ophthalmol Visual Sci. 45:769–775. 14985289 10.1167/iovs.03-1125

[evw100-B3] Barlow RB Bolanowski SJ Brachman ML. 1977 Efferent optic-nerve fibers mediate circadian-rhythms in *Limulus* eye. Science 197:86–89. 867057 10.1126/science.867057

[evw100-B4] Barlow RBJ Ireland L Kass L. 1982 Vision has a role in *Limulus* mating behaviour. Nature 296:65–66. 7063008 10.1038/296065a0

[evw100-B5] Battelle B, 2001 Immunocytochemical localization of opsin, visual arrestin, myosin III, and calmodulin in *Limulus* lateral eye retinular cells and ventral photoreceptors. J Comp Neurol. 435:211–225. 11391642 10.1002/cne.1203

[evw100-B6] Battelle B-A. 2013 What the clock tells the eye: lessons from an ancient arthropod. Integr Comp Biol. 53:144–153. 23639718 10.1093/icb/ict020 PMC4031653

[evw100-B7] Battelle B-A, 2015 Opsins in *Limulus* eyes: characterization of three visible light-sensitive opsins unique to and co-expressed in median eye photoreceptors and a peropsin/RGR that is expressed in all eyes. J Exp Biol. 218:466–479. 25524988 10.1242/jeb.116087 PMC4317242

[evw100-B8] Battelle B-A Kempler KE Harrison A Dugger DR Payne R. 2014 Opsin expression in *Limulus* eyes: a UV opsin is expressed in each eye type and co-expressed with a visible light-sensitive opsin in ventral larval eyes. J Exp Biol. 217:3133–3145. 24948643 10.1242/jeb.107383 PMC4148189

[evw100-B9] Battelle B-A Kempler KE Parker AK Gaddi CD. 2013 Ops1-2, G_q_α and arrestin levels at *Limulus* rhabdoms are controlled by diurnal light and a circadian clock. J Exp Biol. 216:1837–1849. 23393287 10.1242/jeb.083519 PMC4074210

[evw100-B10] Behrens M Krebs W. 1976 Effect of light-dark adaptation on ultrastructure of *Limulus* lateral eye retinular cells. J Comp Physiol. 107:77–96.

[evw100-B11] Brown JE, 1984 Myoinositol polyphosphate may be a messenger for visual excitation in *Limulus* photoreceptors. Nature 311:160–163. 6472474 10.1038/311160a0

[evw100-B12] Burset M Seledtsov IA Solovyev VV. 2000 Analysis of canonical and non-canonical splice sites in mammalian genomes. Nucleic Acids Res. 28:4364–4375. 11058137 10.1093/nar/28.21.4364 PMC113136

[evw100-B13] Calman BG Battelle BA. 1991 Central origin of the efferent neurons projecting to the eyes of *Limulus polyphemus.* Vis Neurosci. 6:481–495. 2069900 10.1017/s0952523800001334

[evw100-B14] Calman BG Lauerman MA Andrews AW Schmidt M Battelle BA. 1991 Central projections of *Limulus* photoreceptor cells revealed by a photoreceptor-specific monoclonal-antibody. J Comp Neurol. 313:553–562. 1783680 10.1002/cne.903130402

[evw100-B15] Cao ZJ, 2013 The genome of *Mesobuthus martensii* reveals a unique adaptation model of arthropods. Nat Commun. 4:2602. 24129506 10.1038/ncomms3602 PMC3826648

[evw100-B16] Chamberlain SC Barlow RB. 1980 Neuroanatomy of the visual afferents in the horseshoe-crab (*Limulus polyphemus*). J Comp Neurol. 192:387–400. 7400403 10.1002/cne.901920212

[evw100-B17] Chamberlain SC Wyse GA. 1986 An atlas of the brain of the horseshoe-crab *Limulus polyphemus.* J Morphol. 187:363–386. 3701873 10.1002/jmor.1051870308

[evw100-B18] Chen P, 2001 A photic visual cycle of rhodopsin regeneration is dependent on RGR. Nat Genet. 28:256–260. 11431696 10.1038/90089

[evw100-B19] Church SH Ryan JF Dunn CW. 2015 Automation and evaluation of the SOWH Test of phylogenetic topologies with SOWHAT. Syst Biol. 64:1048–1058. 26231182 10.1093/sysbio/syv055 PMC4604836

[evw100-B20] Crouch TE Lubin Y. 2000 Effects of climate and prey availability on foraging in a social spider, *Stegodyphus mimosarum* (Araneae, Eresidae). J Arachnol. 28:158–168.

[evw100-B21] Dalal JS Jinks RN Cacciatore C Greenberg RM Battelle BA. 2003 *Limulus* opsins: diurnal regulation of expression. Vis Neurosci. 20:523–534. 14977331 10.1017/s095252380320506x

[evw100-B22] Edgecombe GD Legg DA. 2014 Origins and early evolution of arthropods. Palaeontology 57:457–468.

[evw100-B23] Eriksson BJ Fredman D Steiner G Schmid A. 2013 Characterisation and localisation of the opsin protein repertoire in the brain and retinas of a spider and an onychophoran. BMC Evol Biol. 13:186. 24010579 10.1186/1471-2148-13-186 PMC3851285

[evw100-B24] Feuda R Hamilton SC McInerney JO Pisani D. 2012 Metazoan opsin evolution reveals a simple route to animal vision. Proc Natl Acad Sci U S A. 109:18868–18872. 23112152 10.1073/pnas.1204609109 PMC3503164

[evw100-B25] Feuda R Rota-Stabelli O Oakley TH Pisani D. 2014 The comb jelly opsins and the origins of animal phototransduction. Genome Biol Evol. 6:1964–1971. 25062921 10.1093/gbe/evu154 PMC4159004

[evw100-B26] Fleissner G. 1983 Efferent neurosecretory fibers as pathways for circadian clock signals in the scorpion. Naturwissenschaften 70:366–368.

[evw100-B27] Fleissner G Fleissner G. 1988 Efferent control of visual sensitivity in arthropod eyes: with emphasis on circadian rhythms. Stuttgart: Gustav Fischer Verlag.

[evw100-B28] Fleissner G Heinrichs S. 1982 Neurosecretory cells in the circadian-clock system of the scorpion, *Androctonus australis*. Cell Tissue Res. 224:233–238. 6178512 10.1007/BF00217282

[evw100-B29] Fridmanis D Fredriksson R Kapa I Schioth HB Klovins J. 2007 Formation of new genes explains lower intron density in mammalian rhodopsin G protein-coupled receptors. Mol Phylogenet Evol. 43:864–880. 17188520 10.1016/j.ympev.2006.11.007

[evw100-B30] Giuditta A, 2008 Local gene expression in axons and nerve endings: the glia-neuron unit. Physiol Rev. 88:515–555. 18391172 10.1152/physrev.00051.2006

[evw100-B31] Grbić M, 2011 The genome of *Tetranychus urticae* reveals herbivorous pest adaptations. Nature 479:487–492. 22113690 10.1038/nature10640 PMC4856440

[evw100-B32] Grusch M Barth FG Eguchi E. 1997 Fine structural correlates of sensitivity in the eyes of the ctenid spider, *Cupiennius salei* Keys. Tissue Cell 29:421–430. 18627824 10.1016/s0040-8166(97)80028-6

[evw100-B33] Hanna WJB Horne JA Renninger GH. 1988 Circadian photoreceptor organs in *Limulus*.2. The Telson. J Comp Physiol. a 162:133–140.

[evw100-B34] Hartline HK Wagner HG Ratliff F. 1956 Inhibition in the eye of *Limulus.* J Gen Physiol. 39:651–673. 13319654 10.1085/jgp.39.5.651 PMC2147566

[evw100-B35] Harzsch S, 2006 Evolution of arthropod visual systems: development of the eyes and central visual pathways in the horseshoe crab *Limulus polyphemus* Linnaeus, 1758 (Chelicerata, Xiphosura). Dev Dyn. 235:2641–2655. 16788994 10.1002/dvdy.20866

[evw100-B36] Henze MJ Oakley TH. 2015 The dynamic evolutionary history of pancrustacean eyes and opsins. Integr Comp Biol. 55:830–842. 26319405 10.1093/icb/icv100

[evw100-B37] Hering L Meyer G. 2014 Analysis of the opsin repertoire in the Tardigrade *Hypsibius dujardini* provides insights into the evolution of opsin genes in panarthropoda. Genome Biol Evol. 6:2380–2391. 25193307 10.1093/gbe/evu193 PMC4202329

[evw100-B38] Hwang DS, 2014 Complete mitochondrial genome of the monogonont rotifer, *Brachionus koreanus* (Rotifera, Brachionidae). Mitochondrial DNA 25:29–30. 23488916 10.3109/19401736.2013.775274

[evw100-B39] Jezzini SH Bodnarova M Moroz LL. 2005 Two-color in situ hybridization in the CNS of Aplysia californica. J Neurosci Methods. 149:15–25. 16061289 10.1016/j.jneumeth.2005.05.007

[evw100-B40] Kashiyama K Seki T Numata H Goto SG. 2009 Molecular characterization of visual pigments in Branchiopoda and the evolution of opsins in Arthropoda. Molec. Biol Evol. 26:299–311. 10.1093/molbev/msn251 18984904

[evw100-B41] Katoh K Misawa K Kuma K Miyata T. 2002 MAFFT: a novel method for rapid multiple-sequence alignment based on fast Fourier transform. Nucleic Acids Res. 30:3059–3066. 12136088 10.1093/nar/gkf436 PMC135756

[evw100-B42] Katoh K Standley DM. 2013 MAFFT Multiple Sequence Alignment Software Version 7: improvements in performance and usability. Mol Biol Evol. 30:772–780. 23329690 10.1093/molbev/mst010 PMC3603318

[evw100-B43] Katti C, 2010 Opsin co-expression in *Limulus* photoreceptors: differential regulation by light and a circadian clock. J Exp Biol. 213:2589–2601. 20639420 10.1242/jeb.043869 PMC2905303

[evw100-B44] Kenny N, 2016 Ancestral whole-genome duplication in the marine chelicerate horseshoe crabs. Heredity 116:190–199. 26419336 10.1038/hdy.2015.89 PMC4806888

[evw100-B45] Lisman JE Brown JE. 1972 Effects of intracellular iontophoretic injection of calcium and sodium ions on light response of *Limulus* ventral photoreceptors. J Gen Physiol. 59:701–719. 5025746 10.1085/jgp.59.6.701 PMC2203200

[evw100-B46] Liu K, 2012 SATe-II: very fast and accurate simultaneous estimation of multiple sequence alignments and phylogenetic trees. Syst Biol. 61:90–106. 22139466 10.1093/sysbio/syr095

[evw100-B47] Miller MA Pfeiffer W Schwartz T. 2010 Creating the CIPRES science gateway for inference of large phylogenetic trees Proceeds of the Gateway Computing Environments Workshop; New Orleans p. 1–8.

[evw100-B48] Mori K Saito T Kuramoto T. 2004 Physiological and morphological identification of photosensitive neurons in the opisthosomal ganglia of *Limulus polyphemus*. Biol Bull. 207:209–216. 15616351 10.2307/1543209

[evw100-B49] Nagata T Koyanagi M Tsukamoto H Terakita A. 2010 Identification and characterization of a protostome homologue of peropsin from a jumping spider. J Comp Physiol A. 196:51–59. 10.1007/s00359-009-0493-9 19960196

[evw100-B50] Nathans J Hogness DS. 1983 Isolation, sequence-analysis, and intron exon arrangement of the gene encoding bovine rhodopsin. Cell 34:807–814. 6194890 10.1016/0092-8674(83)90537-8

[evw100-B51] Nilsson DE Kelber A. 2007 A functional analysis of compound eye evolution. Arthropod Struct Dev. 36:373–385. 18089116 10.1016/j.asd.2007.07.003

[evw100-B52] Nolte J Brown JE. 1972 Electrophysiological properties of cells in median ocellus of *Limulus.* J Gen Physiol. 59:167–185. 5058473 10.1085/jgp.59.2.167 PMC2203167

[evw100-B53] Nossa C, 2014 Joint assembly and genetic mapping of the Atlantic horseshoe crab genome reveals ancient whole-genome duplication. GigaScience 3:9. 24987520 10.1186/2047-217X-3-9 PMC4066314

[evw100-B54] Pattengale ND Alipour M Bininda-Emonds OR Moret BM Stamatakis A. 2009 How many bootstrap replicates are necessary? Berlin Heidelberg: Springer, p. 184–200. 10.1089/cmb.2009.0179 20377449

[evw100-B55] Porter M Cronin T McClellan D Crandall K. 2007 Molecular characterization of crustacean visual pigments and the evolution of pancrustacean opsins. Mol Biol Evol. 24:253–268. 17053049 10.1093/molbev/msl152

[evw100-B56] Porter ML, 2012 Shedding new light on opsin evolution. Proc R Soc London B. 279:3–14. 10.1098/rspb.2011.1819 PMC3223661 22012981

[evw100-B57] Regier JC, 2010 Arthropod relationships revealed by phylogenomic analysis of nuclear protein-coding sequences. Nature 463:1079–1098. 20147900 10.1038/nature08742

[evw100-B58] Renninger G, 1997 Phase-shifting and entrainment of a circadian rhythm in *Limulus polyphemus* by ocular and extraocular photoreceptors. Biol Rhythm Res. 28(Suppl):50–68.

[evw100-B59] Rokas A Holland PWH. 2000 Rare genomic changes as a tool for phylogenetics. Trends Ecol Evol. 15:454–459. 11050348 10.1016/s0169-5347(00)01967-4

[evw100-B60] Sacunas RB, 2002 Multiple mechanisms of rhabdom shedding in the lateral eye of *Limulus polyphemus*. J Comp Neurol. 449:26–42. 12115691 10.1002/cne.10263

[evw100-B61] Seaver EC Kaneshige LM. 2006 Expression of ‘segmentation’ genes during larval and juvenile development in the polychaetes Capitella sp I and H-elegans. Dev Biol. 289:179–194. 16330020 10.1016/j.ydbio.2005.10.025

[evw100-B62] Shin JH Richard EA Lisman JE. 1993 Ca2+ is an obligatory intermediate in the excitation cascade of *Limulus* photoreceptors. Neuron 11:845–855. 8240808 10.1016/0896-6273(93)90114-7

[evw100-B63] Smith WC Price DA Greenberg RM Battelle BA. 1993 Opsins from the lateral eyes and ocelli of the horseshoe-crab, *Limulus polyphemus.* Proc Natl Acad Sci U S A. 90:6150–6154. 8327495 10.1073/pnas.90.13.6150 PMC46885

[evw100-B64] Stamatakis A. 2006 RAxML-VI-HPC: maximum likelihood-based phylogenetic analyses with thousands of taxa and mixed models. Bioinformatics 22:2688–2690. 16928733 10.1093/bioinformatics/btl446

[evw100-B65] Stamatakis A. 2014 RAxML version 8: a tool for phylogenetic analysis and post-analysis of large phylogenies. Bioinformatics 30:1312–1313. 24451623 10.1093/bioinformatics/btu033 PMC3998144

[evw100-B66] Stamatakis A Hoover P Rougemont J. 2008 A rapid bootstrap algorithm for the RAxML web servers. Syst Biol. 57:758–771. 18853362 10.1080/10635150802429642

[evw100-B67] Sun H Gilbert DJ Copeland NG Jenkins NA Nathans J. 1997 Peropsin, a novel visual pigment-like protein located in the apical microvilli of the retinal pigment epithelium. Proc Natl Acad Sci U S A. 94:9893–9898. 9275222 10.1073/pnas.94.18.9893 PMC23288

[evw100-B68] Swofford D Olsen GJ Waddell PJ Hillis DM. 1996 Phylogenetic inference In: Hiillis DM Moritz D Mable BK, editors. Molecular systematics. Sunderland (MA)” Sinauer Associates p. 407–514.

[evw100-B69] Waterman TH Wiersma CAG. 1954 The functional relation between retinal cells and optic nerve in *Limulus.* J Exp Zool. 126:59–85.

[evw100-B70] Yamashita S. 2002 Efferent innervation of photoreceptors in spiders. Microsc Res Tech. 58:356–364. 12214302 10.1002/jemt.10143

