# Supplementary material for: Opsin Repertoire and Expression Patterns in Horseshoe Crabs: Evidence from the Genome of Limulus polyphemus (Arthropoda: Chelicerata)
Source: Genome Biol Evol. 2016 Apr 29;8(5):1571–89. doi: 10.1093/gbe/evw100 (PMC4898813; doi:10.1093/gbe/evw100)
Supplement: Supplementary Data [file supp_8_5_1571__index.html]

Opsin Repertoire and Expression Patterns in Horseshoe Crabs: Evidence from the Genome of Limulus polyphemus (Arthropoda: Chelicerata) — Supplementary Data 

# Opsin Repertoire and Expression Patterns in Horseshoe Crabs: Evidence from the Genome of *Limulus polyphemus* (Arthropoda: Chelicerata)

## Supplementary Data

files

- Supplementary Data - zip file
