# Supplementary material for: Opsin Repertoire and Expression Patterns in Horseshoe Crabs: Evidence from the Genome of Limulus polyphemus (Arthropoda: Chelicerata)
Source: Genome Biol Evol. 2016 Apr 29;8(5):1571–89. doi: 10.1093/gbe/evw100 (PMC4898813; doi:10.1093/gbe/evw100)
Supplement: Supplementary Data [file supp_evw100_suppl_data.zip › Supplemental Figure combined 03-24-16.pdf]

Supplemental Figure 1

```
Ops1    : ----MAN---QLSYSS-------------LGWPYQP--NASVVDTMPKEMLYMIHEHWYAFPPMNPLWYSILGVAMIILGIICVLGNGMV :  68
Ops2    : ----MAN---QLSYSS-------------LGWPYQP--NASVVDTMPKEMLYMIHEHWYAFPPMNPLWYSILGVAMIILGIICVLGNGMV :  68
Ops5    : ----MSTGSYFIGNSTAPRS--------SGWWSYDP--GLSVRDTAPENIKHLISDHWSKFPAVNPMWHYLLGLIYIVLGIASLTGQSVV :  76
Ops6    : ----MTG---WNPSHSLGHSLWWGSDNEHFLWMRRHGANVTTTDVLPADMEPLIREYWKQFRVLSPEWFFALGICITALGIISLTGNSVV :  83
Ops7    : ----MVE---WNHTESAGLALSGGSDDRYFLWMRRHSDDTAITNLFPHDMGHLIRDHWRQFQAPSSEWYFAIGVIMLIIELIGLTGNFSV :  83
Ops8    : ----MLD---IISSSSP------------FWWQFSS--NASVVDMVPKDMLHMVHEHWYRFPSLNPLWFQILGVAMIFLGILCICGNGVV :  69
UVops1  : ----MVPENLTFRDNQLD-----------FFFQAEPLQQEIHMNGWNAPKDMFINPYWKQFEAPNPFMHYLLGILYTGLMIVACIGNGIV :  75
UVops2  : MLISNMPEFTTSANWTPD-SEQSKSRDVGLKNSTYTDPVEQWRELLTPEFFELVPDFWLHFPPPSEEAHQTLGVLYTLIMVPGILGNALI :  89
Ops9    : -----MTDSTVLTNWTDGGSEEKRETDFVSTGWAHRSPIDQWRELLSPEYFNIVPDLWLQFPPPSIEAHQVLGAVYTLIMVPGVLGNVLL :  85
Ops10   : -------------------------------NATVVSALDKWKTILTSDFLQLVPNHWLQFDPPSDLSHYILGTVYFVVMVTGILGNGTI :  59
Arthop1 : ------MSLCLDAEDFIRAYIGSSEEMNVSQELRDQSSVFFQSLNSSSSVYHGVFGHWCGYSAITSEIHFVVGSFLLLIGVAGIAGNGLV :  84
Arthop2 : ------MSLCFDAQNFVRTYIGDTKVLNVSVKSLDQSSDFLQHLNSSS-VYHRVFNHWCSYSAISTSAHYFVGILLILVGIAGIGGNGLV :  83
Perops1 : -----------------------------MASSTELGSVDLNMSLGETPEFQADKDTYLVDSTFPTSTHKAVGIYLVIVGILGTFGNGII :  61
Cops2   : ----------------------------------------------------------------------------------------VV :   2
Cops1   : ------------------------------------------------------------------------------------------ :   -

Ops1    : IYLMMTTKSLRTPTNLLVVNLAFSDFCMMAFMMPTMTSNCFA-ETWILGPFMCEVYGMAGSLFGCASIWSMVMITLDRYNVIVRG-MA-A : 155
Ops2    : IYLMMTTKSLRTPTNLLVVNLAFSDFCMMAFMMPTMASNCFA-ETWILGPFMCEVYGMAGSLFGCASIWSMVMITLDRYNVIVRG-MA-A : 155
Ops5    : LYLFAKTKPLRTPANMLIVNLAFSDFMMITQFPVFIINCLGGGAWQLGPLLCEITGFAGGLFGYGSIVTLAVISIDRYNVIVRG-FS-A : 164
Ops6    : IYIFLTTKSLRTPSNLLVVNLAFSDFMMMFTMMPMMSVCCFS-QSWIFGPLACGIYGAFGSGFGSISIISMAIAIDRYRVIVKG-LS-G : 170
Ops7    : IYIFLTTKYLRRPSNMFIVNLAFSDFMMMFTIMPMMVVSCFK-QKWIFGPLMCEIYGACGSGFGSVSIISMVAIAMNRYRVVVKG-LS-S : 170
Ops8    : IYIFLTTKSMRTASNVLIVNLAFSDFMMMGTMMPMMATNCFA-EKWALGPFMCEVYGMLGSLFGCASIWSMVMITYDRYHIIGKG-FS-V : 156
UVops1  : IYVFSMSKTLRTPANLFVVALAVTDFLMMLKT-PVFIYNSFH-AGPVYGNIGCIIYGTVGAYSGLMSAFCNAVISYDRYRVIACP-FS-S : 161
UVops2  : IWLVCSTKTIRTPSNFLVVNLAISDFCMLAKM-PIFIYNSFC-QKPALGVWACQLYGFLGGITGTSAIMTIAAMAFERYYSISRP-LDLS : 176
Ops9    : IWIVCSTKALRTPSHILVVNLAISDFLMMTEI-PLFIYNSFC-QKAAFGVWGCQLYGFIGALTGTTAIMTIAAMAGERYYSISRP-LDPS : 172
Ops10   : VWLFCTVKALRTPSVFLVFNLAVSDLAICLMI-PSFVYNSFS-LGPATGIVGCKMYGFIGGLSGTSAIMTITAMSMERYYSISKP-LGVS : 146
Arthop1 : ILVLTRYRRLRTPANRLLGNLAVSDLLMSCLH-PMASFSSFRHS-WQFGKLGCEFYGSMCGLFGLISITTLSAIALERCLVIAIKPWYSG : 172
Arthop2 : VLVFTRYRRLRTPANRLIVNLAVSDLMMSFLH-FMASYSSFRKS-WQFGKIGCEFYGSLCGLFGLVSIVTLSAIALERCLVIAIKPWYCG : 171
Perops1 : ITMFIRFRTLLTPTNLLLITLAVSDLGIILFGFPFSASSSFA-NRWLFNEGGCQWYAFMGFLFGSAHIGVLALLGLDRYLITCRIDFR-- : 148
Cops2   : ISVILRNKQLRSSMNLILLNMSVCDMTISVTG-TPLTFVAAVYRRWIFGDLVCEVYGFAMSVVGMTQIGTLTAIAVERYIIMSKP--YSS :  89
Cops1   : -----------------------------------------------------------------IGTLTAISTERYIIMSRP--YGS :  21

Ops1    : APLTHKKATLLILFVWIWSGGWTILPFFG-WSRYVPEGNLTSCTVDYLTKDWSSASYVVIYGLAVYFLPLITMIYCYFFIVHAVAEHEKQ : 244
Ops2    : APLTHKKATLLILFVWIWSGGWTILPFFG-WSRYVPEGNLTSCTVDYLTKDWSSASYVIIYGLAVYFLPLITMIYCYFFIVHAVAEHEKQ : 244
Ops5    : SPLTHARSAVFILVIWAWTLGWALPPFFG-WGRYVPEGILNSCSFDYLTRDWATVSYIMGCWICEYALPLMVIIYCYIFIVKAVCDHERH : 253
Ops6    : ERLTYREAVLWICIIWIFVGLWTAAPFFG-WNSYVPEGNLTSCSIDMVSHDWNSRSYVIAYGTSVYLLPLLFLSYTYFYIVKTVVEHEYL : 259
Ops7    : EKLTYKQAILWICLIWIYVGLWTSAPFFG-WSSYILEGNFINCSLDSVSHNWNSRSYVIGYSIGVYFLPLFFLTYTYYYVAKTVIQQESA : 259
Ops8    : SPMTHSKAALMNIFVWAWSIGWTLAPFFG-WNRYVPEGNMTSCTFDYLTKDTHSRSYVVIYATAVYFLPLFITIYCYFFIVRAVADHERS : 245
UVops1  : SKLTNKKAVAMILGIVLYVSPFALLPAFEIWNRYVPEGYLTSCTADYFQHDLNGRSFIFCIWFFAWFIPVIIIFCCYFRIYAAVRDHENQ : 251
UVops2  : GRMTRARASTIVICVWIYAFIFSVLPLFH-VNRYVPEGYLSSCSFDYIARDLTSRLFVLIFFIAAWCVPLVIICVSYCGIILTVRRNQQL : 265
Ops9    : RRMTRARATVIVVCIWIYSFTFSVMPLFR-INQYVPEGYLSSCSFDYLTSDLKSRVYVLVFFIAAWCVPMVIICLSYFGIIFIVRKKQFI : 261
Ops10   : GQANWTLIVCTIAFIWIYSSVFSFIPLFG-INQYVPEGYLNSCSFDYLSEDLASRRFVIAYCFAAWCLPVSIICFCYLGIGFVVRKHQLY : 235
Arthop1 : LHINNSKLCKIVAFIWLYSSVCAVAPLFG-WGNYVPEGFLTSCSFDYLTRTPVNRAYFVFLYILGFFLPLLVILTSYFIIWKAVFQHEQE : 261
Arthop2 : YPITKRKLGRVVAFIWLYCFVCVTPPFLG-WGSYVPEGFLTSCSFDYLTRTPTNRSYFFFLFILGFILPLSVIATSYCVIWKTVVQHERE : 260
Perops1 : RKLTYKRYCQMICAVWVYAIFWSVMPLIG-WGRYGPEPSITTCTIDWRHNDGSYKSFIIVYFVLGFLVPFLLIAICYFNIARQLSVKPVA : 237
Cops2   : TRMTPKRSGLIIACTWFYSLGLCLPPLFG-WSHYVLEPPGIGCSVDWLSTTKNNVSYIIYLFTVGFAIPVSVMVFCYSHIIWKVRKAPRR : 178
Cops1   : SKMSPRRSIFIVICTWLYSLSLCLPPFFG-WSRYVLEPPGISCSVDWMTETKNNKSYIIYLFIAGFFLPVFVMVFCYSQIIRMVQKVPKV : 110

Ops1    : LREQAKKMNVASLRANADQQKQSAECRLAKVAMMTVGLWFMAWTPYLIISWAGVFSSG--TRLTPLATIWGSVFAKANSCYNPIVYGISH : 332
Ops2    : LREQAKKMNVASLRANADQQKQSAECRLAKVAMMTVGLWFMAWTPYLIIAWAGVFSSG--TRLTPLATIWGSVFAKANSCYNPIVYGISH : 332
Ops5    : LREQAKKMNVASLRSNVDTQKASAEMRIAKVALVNVLLWVVSWTPYAAIAMIGIAGDQ--MLITPLRSALPALAGKAASVYNPIVYAISH : 341
Ops6    : LRKQARRMNVDRLKPK--QPGISVEAKISLVALMTVVLWFVAWTPYAALAILGSLTNG--KLVTPLISIWGAVFAKAGSCYNPIVYAISH : 345
Ops7    : LRKYSRCMSVDQLKPR--QSKITVEAKLSMVALITVVMWFVAWTPYIIFIFLGSFTSG--KFFTPLLSLLGAVLAKCASFCDPIVYGVSH : 345
Ops8    : LREQAKKMNVTSLRANGDQQGARAEIRLAKIAMFNVGLWFVAWTPYLIIAFNGIFSDG--TKLTPLATIWGSVFAKANSVYNPIVYGISH : 333
UVops1  : LREQAKKMNVENLRTNQNQKDTRGEIRIAKVAFGIIMLFLFSWVPYIVAFIGAFSTKERQLLTPLMSMVPALTLKASACFDPFIYAINH : 341
UVops2  : FRNTVSQINERSYIQ---KHRNTEIKLAKISVSLISLWMISWTPYAVVALFGISFNY--QLLTPTTSMVPALFCKTASVVDPFLYGLSH : 350
Ops9    : YQNQEQGINLERFNIQ---KHRKNEIKLSKIVFALISFWMISWTPYAMVALLGISFNQ--KLLTPTISMVPALFCKTASVIDPFLYGLSH : 346
Ops10   : LREQARRMNVQNFASS---KQRKTRVQLAKITFCLISLWTLAWTPYAIVALLGVFSRK--DLLLPTVSMGPALFCKFASVIDPFVYGLSH : 320
Arthop1 : MSQIHVNRATSRYITR-----RRSDCKSAEMILCIIGLFLLSWTPYAIVATIGQFWDT--NVVTPWVSAIPSFFAKMSTVYNPIIYGFSH : 344
Arthop2 : MSQASISSISPRFMTR-----KRSDFKSAIMILCVIGLFLLSWSPYAAIATIGQFCNS--SYITPWVSAMPALFAKMSIMYNPIIYGISH : 343
Perops1 : PSLRSAICDQWANERN-----------VTMMCLVIVITFVVSWSPYAIVCLWTVFKPP--STVPSVLTLIPPLFAKASIVFNPIIYLTN : 314
Cops2   : TG-KSHAERAEQRLT--------------LMVATMIISTLTAWTPYAVVSLIMALGFS--HVLGPLSTVSPAIFAKSSVVYNPI------ : 245
Cops1   : TDKRSHAVKAQQRLT--------------LMVAAMVTCALTAWTPYAVVSLIVALGYS--QLIGPLSAVSPAIFAKSCVVYNPIVYFFLN : 184

Ops1    : PRYKAALYQRFP----SLACGSGE-SGSDVKSEASATTTME------------EKPKIPEA----------------------------- : 376
Ops2    : PRYKAALYQRFP----SLACGSGE-SGSDVKSEASATMTME------------EKPKSPEA----------------------------- : 376
Ops5    : PKFRLAMQKEIP------CCCINEPQPQSDTSSEMSTKTSV------------ATVNGEDSTAGGTTNN--------------------- : 392
```

```
Ops6     : PKYQEALRKRLPRLYKFFSCTSSKPLEKVENQENQHESTTI------------EKIISPESSP--------------------------- : 396
Ops7     : PKYREALRERMLKFYKFFSCGKCRPTKENEDHKNQHELTTI------------EKNNSTTEADH-------------------------- : 397
Ops8     : PKYRVALKAKLP----WLFCDTDN-DENFSNESNDTSTLIT------------EKIQLPFKIST-------------------------- : 380
UVops1   : PKYRLELQKKIP-----WLCIHESYSDNASTCSNKTQLSGD------------TTPTVNSDG---------------------------- : 386
UVops2   : PRFKSELKKKIICLYVLNKFAKQKESFNIPIPSHVSDESSFSLQYPSLKQKLEEPLGHQVSTISRCHQDIFLTDSQSLPPNILYTGIEHQ : 440
Ops9     : PRFKAALKKKVSCLHITNKNTKRKRSFNIHSFQHASLES-------------RESLDRQLPPINRYRGDSSETSFQNISLTIIHPQKGRP : 423
Ops10    : PRFKKELKKN----------------------FHGSL----------------------------------------------------- : 335
Arthop1  : REFCACTRHLFTRTQTPALRKKIYSRYSQNTPR-HEKQTRLSRASG------VEKLNNDVKQQPELKNKFRKCYIVMSFLQEGRSMKTVS : 427
Arthop2  : RRFCSCIRLLFMKTQIPPPKKKIYMRFSKGLPRRDNRQTFLSTACG------DYRVRGDVSLQP--TNKGRKCYVMMSLGREGKSTNTVF : 425
Perops1  : PRLRMGIIATITCSGELPGEMIPVSSNPEATPETHESI---------------------------------------------------- : 352
Cops2    : ------------------------------------------------------------------------------------------ :   -
Cops1    : PQIQEATMETFRRNRPDLQSALQQDISLIATNNYTDVTRRSPVTNN------LLSLRSDRTQSFL------------------------- : 243


Ops1     : ------------------------------------------------------------------------------------------ :   -
Ops2     : ------------------------------------------------------------------------------------------ :   -
Ops5     : ------------------------------------------------------------------------------------------ :   -
Ops6     : ------------------------------------------------------------------------------------------ :   -
Ops7     : ------------------------------------------------------------------------------------------ :   -
Ops8     : ------------------------------------------------------------------------------------------ :   -
UVops1   : ------------------------------------------------------------------------------------------ :   -
UVops2   : KHKRFHSVSCYLNESCEKPLEMGVFSNH-------------------------------------------------------------- : 468
Ops9     : KRK-------YSNS----------FSNH-------------------------------------------------------------- : 434
Ops10    : ------------------------------------------------------------------------------------------ :   -
Arthop1  : LNQK-------------------------------------------------------------------------------------- : 431
Arthop2  : AEPQAEHCEKLTCDLCLLEATAHKYQRNMWVRKLLSDSSIYSRSKANVNNDLSLPECLIKQKYRYEACFCWYDHKNSSICYNTTCSCSCE : 515
Perops1  : ------------------------------------------------------------------------------------------ :   -
Cops2    : ------------------------------------------------------------------------------------------ :   -
Cops1    : ------------------------------------------------------------------------------------------ :   -


Ops1     : ------------------------------------------ :   -
Ops2     : ------------------------------------------ :   -
Ops5     : ------------------------------------------ :   -
Ops6     : ------------------------------------------ :   -
Ops7     : ------------------------------------------ :   -
Ops8     : ------------------------------------------ :   -
UVops1   : ------------------------------------------ :   -
UVops2   : ------------------------------------------ :   -
Ops9     : ------------------------------------------ :   -
Ops10    : ------------------------------------------ :   -
Arthop1  : ------------------------------------------ :   -
Arthop2  : FRLEASPSSAKKEHYIKRPSGGSLPARCASCYYCDRKIIHYY : 557
Perops1  : ------------------------------------------ :   -
Cops2    : ------------------------------------------ :   -
Cops1    : ------------------------------------------ :   -
```

-

Supplemental figure 1. Clustal W alignment of predicted amino acid sequences of confirmed *Limulus* opsin transcripts. Positions of the transmembrane domains, indicated by lines above the sequences, are estimated from an alignment with bovine rhodopsin (Palczewski et. al., 2000). The following amino acids are highlighted: cyan, lysine (K) at the site equivalent Gly^{90} in bovine rhodopsin responsible for determining UV sensitivity in rhabdomeral opsins highlighted; green, two cystines (C) that are conserved in all opsins; yellow, an indel highly conserved in many arthropod opsins (Porter et al., 2007); purple, triplet of amino acids characteristic of R-opsins coupled to $G_{q/11}$; red, amino acids containing a splice site. If the intron is located between two amino acids both amino acids are highlighted in red. Also annotated are the conserved lysine that is the chromophore binding site (♦) and two potential sites for the Schiff base counter-ion equivalent to sites 83 and 181 in bovine rhodopsin (*). Amino acids highlighted in black are conserved in all 15 sequences; those highlighted in dark gray are conserved in 12-14 and those in light gray are conserved in 9-11.

**Ops1**	**Ops1**															
**Ops2**	99/100	**Ops2**														
**Ops5**	45/61	46/63	**Ops5**													
**Ops6**	46/65	47/64	39/57	**Ops6**												
**Ops7**	38/58	38/58	33/53	61/77	**Ops7**											
**Ops8**	65/79	66/79	45/63	45/61	39/57	**Ops8**										
**UVOps1**	36/54	34/51	38/53	31/47	25/45	36/53	**UVOps1**									
**Ops9**	25/48	24/45	29/49	27/45	25/46	27/49	27/49	**Ops9**								
**Ops10**	31/49	32/49	29/48	28/44	24/43	32/50	32/50	41/53	**Ops10**							
**UVOps2**	26/47	26/47	27/45	25/42	23/42	27/48	27/48	59/70	37/50	**UVOps2**						
**ArthOps1**	26/44	25/42	27/44	24/38	24/40	24/41	24/41	28/48	25/42	26/44	**ArthOps1**					
**ArthOps2**	21/35	21/35	23/34	20/34	21/36	20/34	20/34	20/34	21/31	20/34	50/60	**ArthOps2**				
**COps1**	15/29	17/31	20/30	15/28	15/27	17/29	17/29	17/29	17/29	14/26	18/30	14/22	**COps1**			
**COps2**	20/35	19/35	20/36	20/34	19/32	20/33	20/33	20/33	23/38	15/29	21/32	15/25	52/60	**COps2**		
**PerOps1**	28/50	29/51	24/44	22/42	22/42	24/45	23/41	24/45	24/39	21/39	24/38	18/30	15/26	20/37	**PerOps1**	
**PerOps2**	22/41	23/41	21/39	24/41	21/39	23/40	23/40	23/40	23/39	19/36	22/38	19/30	18/29	19/32	58/67	**PerOps**

Supplemental Figure 2

Comparisons of identities/similarities among the predicted amino acid sequences of *Limulus* opsins. Identities/similarities were determined using EMBOSSNeedle pair-wise comparisons. Opsin pairs with 50% or greater amino acid identities are highlighted in yellow; those with 40% or greater amino acid sequence identities are highlighted in green.

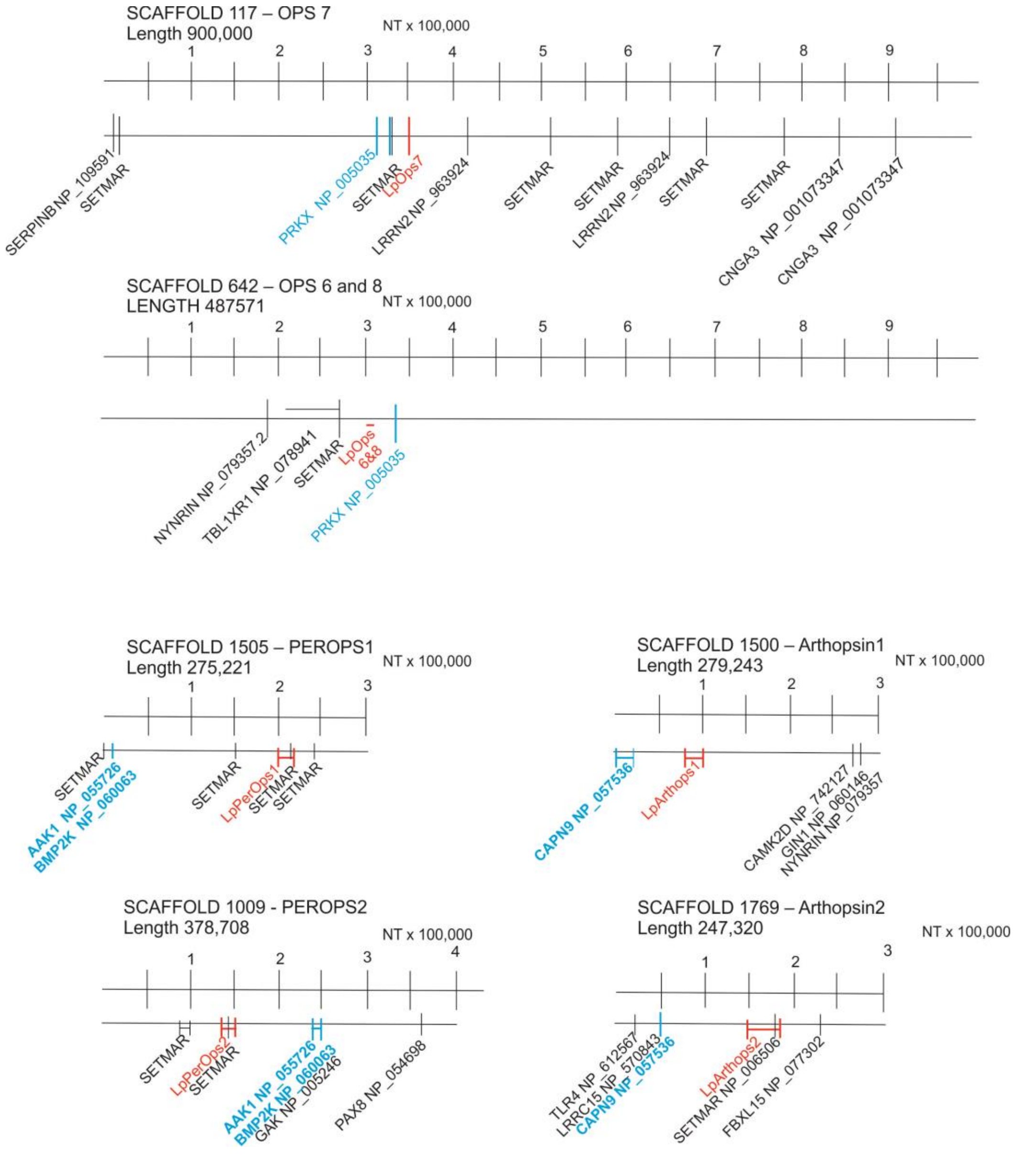

Supplemental Figure 3. **Scaffolds encoding *Limulus* opsin paralogs LpOps7 and 6 and 8, LpPerOps1 and 2, and LpArthops1 and 2 encode other shared genes**. Sequences of scaffolds encoding *Limulus* opsin paralogs were BLASTed against the human reference genome. In the representation of each scaffold, the upper line shows its length marked off in 1kb nucleotide lengths. The positions of all other identified genes are shown on the lower line. The opsin genes are in red; other genes shared on scaffolds encoding opsin paralogs are in blue. Genes with a best hit to human SETMAR transposases were found on most scaffolds examined and are considered non-specific.

```
                          *        20         *        40         *        60         *        80
LpOps10     : ------------------------------------------------------------------------------NA   2
Mite        : ------------------------------------MGTRESKMMSSSISGLAASSSTLTASASRLANDRLAPSTKSIYY  44
Daphnia     : ---------------------------------------------------MCNQTNCATDVVTDDLNCPWLAEYQLRHS  29
Mosq-UV7    : --------------------------------------------------------------------------------   -
Capitella1  : ---------------------------------------------MSYADGYLDNSTAPIEESPYLPHGTFFHPHWRPYR  35
Capitella2  : -----------------------------------------------------------------MTLTPAIQPNLNRES  15
Urchin-Mel  : -------------------------------------------------------MNAVTTALPHGLNKPTIEARWT---  22
Mouse-Mel   : -----------------------------------------------MDSPSGPRVLSSLTQDPSFTTSPALQGIWNGTQ  33

                          *       100         *       120         *       140         *       160
LpOps10     : TVVSALDKWKTILTSDFLQLVPNHWLQFDPPSDLSHYILGTVYFVVMVTGILGNGTIVWLFCTVKALRTPSVFLVFNLAV   82(2-1)
Mite        : CDHDKGAIWSDLLTTDLLEYIPDHWSQFEPPSSCTNLLLAILYFLIFVFGCGGNGLVIVLYLKNKKLRSPSNLILFNLAI  124(2-1)
Daphnia     : AIIERWKLCGFFEDDDFLDIN-CHWLQFEPAPLVNHLLLVCIFVFILLTGCLCNIIVIYIFASSRHLRTPANIIIMNLAI  108(2-1)
Mosq-UV7    : -----------------MRKWN---------------------------------------AQFKSLRTPANYLVINLAI  124(2-1)
Capitella1  : EMLLNMN-------------------------PLIYYGLGLYMAVVGIVGTLGNLVVITLFIKTRSLRTPPNMFIINLAL   90(2-1)
Capitella2  : ----------------------------------HSLIGACLIIILFVLAMVGNGLIIFAFLRFQDLRIQCYILIVNLAV   60(2-1)
Urchin-Mel  : ----------------------------------------------------------------KSLRTPPNMLIVNLAI   38
Mouse-Mel   : NVSVRAQLLSVSPTTSAHQAAAWVPFPTVDVPDHAHYTLGTVILLVGLTGMLGNLTVIYTFCRNRGLRTPANMFIINLAV  113(2-1)

                          *       180         *       200         *       220         *       240
LpOps10     : SDLAICLMI---PSFVYNSFSLGPATGIVGCKMYGFIGGLSGTSAIMTITAMSMERYYSISKP-LGVSGQANWTLIVCTI  158(1-2)
Mite        : ADFFMIFKT---PVFIYNSVKCGPALGSLGCDVYGFLGGLTGCGAIFSVAIASIDRYNVISNP-LNP-RRLTTVTSTLAV  199(1-2)
Daphnia     : SDFFMLIKM---PIFLYNSLLQGPALGIKGCQIYGFMSGLTGTTSIMSLATVAVDRYLVISQP-LNVNRKSTRTRAYLTT  184(1-2)
Mosq-UV7    : ADFIIMLEA---PLFVYNSYHQGPATGNVWCTIYALLGAVGGTVAIVTLTMISIDRYNVVVYP-LNPKRSTTRLKVALMI  100(1-2)
Capitella1  : SDMGFCATNG-FPLMTVASFQKLWRWGPVACELYALAGSITGFNSIATLALISMDRYMVIAKP-FYAMKHVSHKRSLIQI  168(1-2)
Capitella2  : ADFLMTTLG--LPLTIYSAFSPRWPFSNSVCQLYGFSGGLFGLTSINTQAAIAIDRCLVLIRSQKAQPVRRSHRQTAFVV  138(1-2)
Urchin-Mel  : SDFGMVITN--FPLMFASTIYNRWLFGDAGCQFYAFCGALFGIMSIANMTAIALDRYYVICWS-LEAVRSVTHRRSMIII  115(1-2)(2-1)
Mouse-Mel   : SDFLMSVTQ--APVFFASSLYKKWLFGETGCEFYAFCGAVFGITSMITLTAIAMDRYLVITRP-LATIGRGSKRRTALVL  190(1-2)

                          *       260         *       280         *       300         *       320
LpOps10     : AFIWIYSSVFSFIPLFGIN--QYVPEGYLNSCSFDYLSEDLASRRFVLAYCFAAWCLPVSIICFCYLGIGFVVRKHQLYL  236
Mite        : LFIWAYSAFFATLPLLQIFN-RYTYEGFLTSCTVDYLAEETSVRAFVFCYFAAAWCVPVTIIVCSYYGIYKAVGASEKSL  278
Daphnia     : CFIWLYSAIFASLPLFGIG--KYVPEGYLTSCSFDYLSNNVKTRSFILAFFLAAWLFPFCIITTCYTAIVWYVRKARIEL  262
Mosq-UV7    : VFAWIYGLVFSVIPALDIGLSRYTPEGFLTACSFDYLERTRDARLFMFLYFIFAWVVPIIAITFCYIQILRVVIGANSIQ  180
Capitella1  : ILAWTWAFIWSAPPLLRMGYGRYIPEGFQVSCTFDYLSRDLKNLIFVWCLFVFGFFIPVLAIACSYVGIIRAVGAQSKEM  248
Capitella2  : AIPWLYAFLWSVPPLYGWN--GYRLDELQTICSFDIVAESFSSRVFVVCVGVFGFLLPLCIIIFCYSFIFVAINKYKKGF  216
Urchin-Mel  : IIVWCYAIFWSIPPFF--GVGSYVLEGYGLGCTFDFMTKDLNHYLHVSFLFASSFVVPVTIIIVCFTRIAITVRAHRHEL  193
Mouse-Mel   : LGVWLYALAWSLPPFF--GWSAYVPEGLLTSCSWDYMTFTPQVRAYTMLLFCFVFFLPLLIIIFCYIFIFRAIRETGRAC  268(1-2)(2-1)

                          *       340         *       360         *       380         *       400
LpOps10     : REQARRMN-------VQNFASSKQRKTRVQLAKITFCLISLWTLAWTPYAIVALLGVFSRK--DLLLPTVSMGPALFCKF  307
Mite        : RKIEDTTGGT-----SGKDKGKNSQKEEIKIAKTAAVLISLWLVSWTPYAAIALTGATGNG--ALLNPGWSMIPALTAKI  351
Daphnia     : NHQQAAIT-------NCKRG-WRQCNIEVVIAKIVVVLVAMWMISWTPYAFVALLGISGTQ--TRLTPGMTMFPALFAKF  332
Mosq-UV7    : SSK-------------------NKSKTEVKLAGVVIGIIGLWFIAWTPYAIVAMMGVFGYE--SLLSPLGSMVPAILAKT  239
Capitella1  : RKTAEKMGAKTG-------KSDKEKKQDIAMAKVAAGTIGLFLMSWTPYAAVSMIGIAGNR--SWITPYVSQIPVMFAKA  319
Capitella2  : YEISESLRFSVYIG----SAVVPHPKRDYKTAQIGLTCVIFFCLAWMPYAVVGWISVAGYY--NKVGPHVLLMTTLFAKT  290
Urchin-Mel  : NKMRTKLTEDKDKKHKSSIRRANKAKTEFQIAKVGFQVTIFYVLSWMPYSIVAVIGQYFDS--DLLTPLGTVVPVIFAKC  271
Mouse-Mel   : EGCGESPLRQR--------RQWQRLQSEWKMAKVALIVILLFVLSWAPYSTVALVAFAGYS--HILTPYMSSVPAVIAKA  338(2-1)

                          *       420         *       440         *       460         *       480
LpOps10     : ASVIDPFVYGLSHPRFKKELKKN----------------------FHGSL------------------------------  335
Mite        : AATVDPFVYSLSHPKFKKEIEAMFG-------------------------------------------SKSQGKGAANKA  388
Daphnia     : SACVNPIIYTLTHPKIKKETLRRWYCFMSSGTSNGNDVTLNGGGVSVGRQDPDWRQNSNSDKGSVSPVAHHHNSKNFSAN  412
Mosq-UV7    : AACIDPYFYAMNHPRYRQELRKMFG---LNQQDLGNSQYQTSRYTRNASRMDDSEGGASERVTIGRQPGKTTTDEPEPSQ  316
Capitella1  : SAMWNPIIYALSHPKFRAALEDHMP----WLLVC----------------------------------------------  349
Capitella2  : STIYNPTIYAACLPRFRRRVQYLFQS-----------------------------------CCKPKHRHIQSRSYSLNVS  335
Urchin-Mel  : SAIWNPIIYCLSHEKFNAALKEKL------MGMCG------IEIPSKHRSMGSQESSVTGRRGMHRQNSSTLSESSVTST  339
Mouse-Mel   : SAIHNPIIYAITHPKYRVAIAQHLPCLGVLLGVSGQRSHPSLSYRSTHRSTLSSQSSDLSWISGRKRQESLGSESEVGWT  418(2-1)
```

```
                        *       500         *       520         *       540         *       560
LpOps10    : --------------------------------------------------------------------------------   -
Mite       : PADDTATRSAIWPSMVH--------------------------------------------------------------- 405
Daphnia    : KNDPTGSDIQPQELVPLNDHSDRGDNSLQTACQNCTDKTGNSYMDERLSYNETCFSVIAPESIDLYKTKVMSVHNDNLQN 492
Mosq-UV7   : QTEQGPQPTYSKNLAANSRGALQRAQSSISAADDTSLSVSIDLTETNPNSNH---------------------------- 368
Capitella1 : --------------------------------------------------------------------------------   -
Capitella2 : KEHSIPMNNLSWEVVLSPDVSGKRSQGYHTQRSWGEIDTQQSSTRFRNMTHAVKDSGCGISRV----------------- 398
Urchin-Mel : VDQDAIELKDRKQGPATVKVQQEKVEGGTYRRNPGDVTFSKDAGVEVDEKRR--GDQGQRDDRVRPQGEGQMDQWS--QP 415
Mouse-Mel  : DTETTAAWGAAQQASGQSFCSQNLEDGELKASSSPQVQRSKTPKVPGPSTCRPMKGQGARPSSLRGDQKGRLAVCTGLSE 498

                        *       580         *       600         *       620         *       640
LpOps10    : --------------------------------------------------------------------------------   -
Mite       : --------------------------------------------------------------------------------   -
Daphnia    : PCLGRYLDLFKDPPINVSGNSISSSDTFEQSKEECSTNIQNNDALLYDKN------------------------------ 542
Mosq-UV7   : -------------------------------------------------------------------------------- : -
Capitella1 : -------------------------------------------------------------------------------- : -
Capitella2 : -------------------------------------------------------------------------------- : -
Urchin-Mel : PPAPASASAPTPGVNDKEYLTKM--------------------------------------------------------- 438
Mouse-Mel  : CPHPHTSQFPLAFLEDDVTLRHL--------------------------------------------------------- 521
```

Supplemental Figure 4 . The locations and phases of the two introns identified in LpUVOps1, LpUV7 opsins and LpArthopsins are conserved in some opsins from a wide variety of species. The opsins were aligned with Clustal W. The opsins used in this alignment are: LpOps10, *L. polyphemus*, KU40435; Mite, *T. urticae* tetur24g02280.1; *D. pulex*, EFX70796, UNOP2 ; Mosquito UV7, *A. aegypti*, AAEL005322-PA; Capitella1, *C. teleta*, jgi|Capca1|119596; Capitella2, *C. teleta*, jgi|Capca1|124377; Urchin-Mel, *S. purpuratus,* XM 011670231; Mouse-Mel, *Mus musculus*, melanopsin4, EU303118. The amino acids containing the splice site are highlighted in green. The phases of the introns are shown at the right of the alignment in parentheses. Amino acids highlighted in black are conserved in all eight sequences, those in dark gray are conserved in six or seven and those in light gray are conserved in five or six.

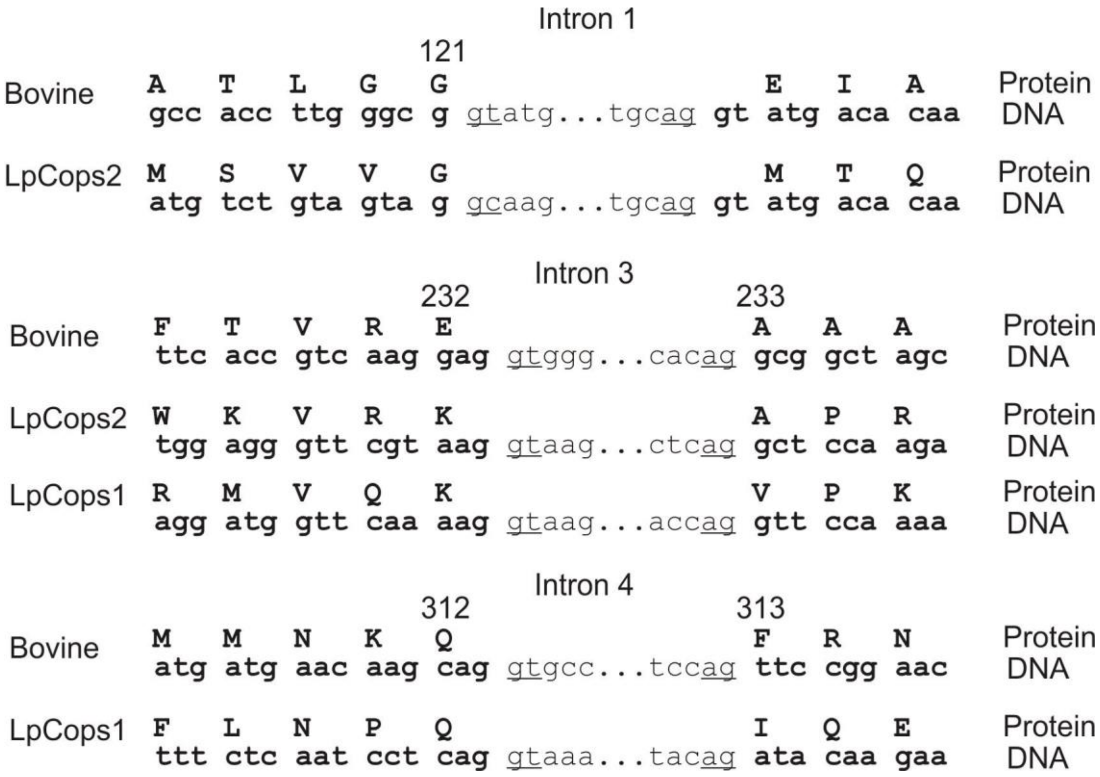

Supplemental Figure 5. The three introns in *Limulus* C-opsins align in position and phase with introns 1, 3 and 4 of bovine rhodopsin. The donor sequence in the first intron of LpCops2 is *gc* instead of the canonical *gt*. Amino acid and nucleotide sequences surrounding splice sites in bovine rhodopsin and *Limulus* C-opsins are shown. Amino acid sequences are in upper case bold letters, nucleotide sequences of exons in lower case bold letters and nucleotide sequences of introns in lower case. Donor and acceptor sequences of the introns are underlined. Amino acid numbering is based on bovine rhodopsin.

```
                      *         20         *         40         *         60         *         80
LpOps1     : ---------------MANQLSYSSLGWPYQP-------NASVVDTMPKEMLYMIHEHWYAFPPMNPLWYSILGVAMIILGI :  59
ManducaRh1 : ------------MD-PGPGLAALQAWAAKSP--AYGAANQTVVDKVPPDMMHMIDPHWYQFPPMNPLWHALLGFTIGVLGF :  66
PapilioRh3 : ---------MALDYLNTGAAKMGTWNGQMS--AYG-ANQTVVDKVLPEMLHLIDPHWYQFPPMNPLWYGLLGFTIACLAI :  68
DrosRh1    : ---------------MDSFAAVATQLGPHFA----ALSNGSVVDKVTPDMAHLISPYWNQFPAMDPIWAKILTAYMIIIGM :  62
ApisRh2    : MDTLNITTSFFIEVMPSNISTLTTTGPQFARQLMRFNNQTVVSKVPEEMLHLIDLYWYQFPPLDPLWHKILGLVMIILGI :  80

                      *        100         *        120         *        140         *        160
LpOps1     : ICVLGNGMVIYLMMTTKSLRTPTNLLVVNLAFSDFCMMAFMMPTMTSNCFAETWILGPFMCEVYGMAGSLFGCASIWSMV : 139
ManducaRh1 : VSISGNGMVIYIFMSTKSLKTPSNLLVVNLAFSDFLMMCAMSPAMVVNCYYETWVWGPFACELYACAGSLFGCASIWTMT : 146
PapilioRh3 : TSITGNAMVIYIFTTTKNLKTPSNLLVVNLAVSDFLMMACMAPPLIINSYNETWVFGPLFCAIYACGGSLYGTVSIWTMT : 148
DrosRh1    : ISWCGNGVVIYIFATTKSLRTPANLLVINLAISDFGIMITNTPMMGINLYFETWVLGPMMCDIYAGLGSAFGCSSIWSMC : 142
ApisRh2    : MGWCGNGVVVYVFIMTPSLRTPSNLLVVNLAFSDFIMMGFMCPPMVICCFYETWVLGSLMCDIYAMVGSLCGCASIWTMT : 160

                      *        180         *        200         *        220         *        240
LpOps1     : MITLDRYNVIVRGMAAAPLTHKKATLLLLFVWIWSGGWTILPFFGWSRYVPEGNLTSCTVDYLTKDWSSASYVVIYGLAV : 219
ManducaRh1 : MIAFDRYNVIVKGIAAKPMTSNGALLRILGIWVFSLAWTLLPFFGWNRYVPEGNMTACGTDYLSKSWVSRSYILIYSVFV : 226
PapilioRh3 : AIAFDRYNVIVKGIAAKPMSINGALLRILAIWLSSLAWTVAPIFGWNRYVPEGNMTVCGTDYLSKDMLSRSYIIAYAVFC : 228
DrosRh1    : MISLDRYQVIVKGMAGRPMTIPLALGKIAYIWFMSSIWCLAPVFGWSRYVPEGNLTSCGIDYLERDWNPRSYLIFYSIFV : 222
ApisRh2    : AIALDRYNVIVKGMSGTPLTIKRAMLQILGIWLFGLIWTILPLVGWNRYVPEGNMTACGTDYLSQDWTFKSYILVYSFFV : 240

                      *        260         *        280         *        300         *        320
LpOps1     : YFLPLITMIYCYFFIVHAVAEHEKQLREQAKKMNVASLRANADQQKQSAECRLAKVAMMTVGLWFMAWTPYLIISWAGVF : 299
ManducaRh1 : YFLPLLLIIYSYFFIVQAVAAHEKAMREQAKKMNVASLRS-SEAANTSAECKLAKVALMTISLWFMAWTPYLVINYTGVF : 305
PapilioRh3 : YFLPLGLIIYSYWFIIQAVAAHEKAMREQAKKMNVASLRS-SDAANTSAECKLAKVALMTISLWFMAWTPYLVINFAGVF : 307
DrosRh1    : YYIPLFLICYSYWFIIAAVSAHEKAMREQAKKMNVKSLRS-SEDADKSAEGKLAKVALVTISLWFMAWTPYLVINCMGLF : 301
ApisRh2    : YYTPLFTIIYSYYFIVSAVAAHEKAMKEQAKKMNVTSLRS-GDNQNTSAEAKLAKVALTTISLWFMAWTPYLVINYIGIF : 319

                      *        340         *        360         *        380         *
LpOps1     : SSGTRLTPLATIWGSVFAKANSCYNPIVYGISHPRYKAALYQRFPSLACGSGESGSDVKSEASATTTMEEKPKIPEA : 376
ManducaRh1 : ES-APISPLATIWGSLFAKANAVYNPIVYGISHPKYQAALYAKFPSLQCQSAPEDAGSVASGTTAVSEEKPAA---- : 377
PapilioRh3 : ET-APISPVSTIWGSVFAKANAVYNPIVYGISHPKYRAALYQRFPSLACQPSPDESGSVASGNTAVCEEKPPA---- : 379
DrosRh1    : KF-EGLTPLNTIWGACFAKSAACYNPIVYGISHPKYRLALKEKCPCCVFGKVDDGKSSEAQSQATNSEAESKA---- : 373
ApisRh2    : NR-SLITPLFTIWGSLFAKANAIYNPIVYGISHPKYRAALKEKLPFLVCG------STEDQTAATAGDKASEN---- : 385
```

Figure 6. Splice sites in LWS LpOps1 align with those in LWS opsins from some insects. The following opsins were aligned with Clustal W: LpOps1, *Limulus polyphemus*, AAA02499; *Manduca sexta* Rh1, AAD11964; *Papilio glaucus* Rh3, AAD29575.1; *Drosophila melanogaster* Rh1, P28678.4; *Apis mellifera*, Rh2, NP_001071293.1. Amino acids containing a splice site are in red. If the intron is located between two amino acids both amino acids are highlighted in red. Amino acids highlighted in black are conserved in all five sequences, those in dark gray are conserved in four and those in gray are conserved in four, those in light gray are conserved in three.

A.

```
                    *        20         *        40         *        60         *        80
EFX77475 : MSSSAINETLAPAAFTSFTQGQK-TFDPWSVPDSFTVSVFAPDYVKPLLHPHWETQKAVHPLWAYFFGLYYLVMGSMAIC :  79
EFX87234 : ----MENITMNAGRNVSLAMAQKHDFNPWTVPDDYTVYNFAPDYVKPLLHPHWQTQKAVHPIYAYFFGMYYLVMGSCAIF :  76
EFX77471 : ----------MELLSNVTAMAQQQPFNPWSLPDTFTIYNFAPEEIRSFLHPHWHNQKAPHPMMYYFFGIIYLVIGTVAIG :  70
EFX77474 : ------------MATNVTAMAQQQPFNPWVLPDSFTVYAFAPEEIRSFLHPHWQTQKAPHPMIYYLFGILYLVIGVMATG :  68
LpOps5   : ---------MSTGSYFIGNSTAPRSSGWWSYDPGLSVRDTAPENIKHLISDHWSKFPAVNPMWHYLLGLIYIVLGIASLT :  71

                    *       100         *       120         *       140         *       160
EFX77475 : GNVMVLKIFSHFKCLRTPANMLVINLAVSDLMLMLSLIPESVYNFFLGGPWQFGHIGCQIHSFCGAFFGYSQITTLTIIS : 159
EFX87234 : GNILVLKIFSRYKSLRTPANMLVMNLAVSDLLLMFSLIPEAVYNFFCGGPWQFGDLGCQIHAFCGAFFGYSQITTLTIIS : 156
EFX77471 : GNWMVLRILGSFPALRTPANMLVMNLAVSDFLLMITLIPECVYNFFTGGPWQFGDLGCQIHAFCGALCGYSQITTLVLIS : 150
EFX77474 : GNYMVLRILGSCPALRTPSNMLVMNLAVSDFLLMITLLPEACYNFFTGGPWRFGDLGCQIHAFCGAICGYSQITTLVLIS : 148
LpOps5   : GQSVVLYLFAKTKPLRTPANMLIVNLAFSDFMMMITQFPVFIINCLGGGAWQLGPLLCEITGFAGGLFGYGSIVTLAVIS : 151

                    *       180         *       200         *       220         *       240
EFX77475 : WDRYNVIVKGFSGKPLTYAKVSLLILFNWLWAFGWALAPLVGWGLYALDGMLGTCSFDSYTTTMNHKSYILASCFFQFAG : 239
EFX87234 : WDRYNVIVNGLKGTPLTFAKAATLILFNWIWSFGWALAPLVGWGLFALDGMLGTCSFDSYTTTMNHKSYILASCFFQYGG : 236
EFX77471 : YDRYNVIVKGFNANPLTFSKAIVFITLTWIWAFGWSVGPLVGWGYYAMDGMLGTCSFDGVTTNISNKSHILTCFTFMFVF : 230
EFX77474 : WDRYNVIVKGFNAAPLTFGKASTFIILSWLWSFGWSVCPLVGWGYYAMDGMLGTCSFDGVTTDMSNKTHILATTLAIYVI : 228
LpOps5   : IDRYNVIVRGFSASPLTHARSAVFILVIWAWTLGWALPPFFGWGRYVPEGILNSCSFDYLTRDWATVSYIMGCWICEYAL : 231

                    *       260         *       280         *       300         *       320
EFX77475 : PIIVIVGCYFFIVKAVFNHEEELRGQAKKMNVTSLRSVNKEQELAVSAEIRAAKVAVVNIL-LWIFAWAPFTTVAMVGTW : 318
EFX87234 : PIIVIVGCYFFIVKAVFHHEEELRVQAKKMNVTSLR--NNAEQDAVTAEMRIAKIAIINIT-LWIFAWTPFTAISMIGTW : 313
EFX77471 : PVLIIISCYYFIVRAVFHHEDELRQQAKKMNVTSLR--SNSEQQAVSAEIRIAKVAIINVT-LWIMAWSPFAVVCLIGTW : 307
EFX77474 : PVIIIIGCYYFIVRAVFHHEDELRQQAKKMNVTSLR--SNSDQQAVSAEIRIAKVACINVT-LWVLAWTPFAAVCMLGTW : 305
LpOps5   : PLMVIIYCYIFIVKAVCDHERHLREQAKKMNVASLR--SNVDTQKASAEMRIAKVALVNVL-LWVVSWTPYAAIAMIGIA : 308

                    *       340         *       360         *       380         *       400
EFX77475 : YDASFVTPLMSELPIICAKTSALYNPVIYALSHPKYRECLKELYPWLCIMVDDRKTVRGRNGDSSSAVTSIKTETSSE-- : 396
EFX87234 : ISPTYITALVSEVPIVCAKTSAVYNPIIYALSHPKYRECLKEICPWLCIVIEKKKGP-NYSSDSDSAKTSATDSA----- : 387
EFX77471 : GDVSRITPLVCELPVILSKTSCAYNPVIYALSHPKYRECLKELYPWLCIVVDTKKSSSKQGGDDQSTNSTKTEASATTQG : 387
EFX77474 : GDTSMITPLICELPVILAKTSCVYNPIIYALSHPKYRECLKDLYPWLCIVVENKK-GKKHGGDDQSTNSTKTEDSGTTQG : 384
LpOps5   : GDQMLITPLRSALPALAGKAASVYNPIVYAISHPKFRLAMQKEIPCCCINEPQPQSDTSSEMSTKTSVATVNGEDSTAGG : 388

EFX77475 : ---- :   -
EFX87234 : ---- :   -
EFX77471 : ---- :   -
EFX77474 : ---- :   -
LpOps5   : TTNN : 392
```

B.

```
                      M   L   G   T                        C   S   F   D   Protein
Daphnia EFX77471:     atg ctc gga ac gtaaggc----aaaaaag a tgc tcc ttt gat DNA
                      I   L   N   S                        C   S   F   D   Protein
LpOps5:               att ctt aat ag gtaggtt----tttctag c tgt agc ttt gac DNA
```

Supplemental Figure 7. Intron 2 of LpOps5 matches in position and phase with intron 4 of opsins from *D. pulex* that cluster within the same subclass in the larger clade of MWS opsins. A. Alignment of LpOps5 with representative *D. pulex* opsins from the same subclass. The amino acids containing splice sites are shown in red. When the splice site is between amino acids, the amino acids on either side of the intron are highlighted. Inverted triangle: location of the intron conserved in LpOps5 and the *D. pulex* opsins. Amino acids highlighted in black are conserved in all five sequences, those in dark gray are conserved in four sequences and those in light gray are conserved in three sequences. B. Alignment of the splice site conserved in the *D. pulex* opsins and LpOps5 to show the phases of the introns. The sequence of a representative *D. pulex* sequence is shown. The amino acid sequences are shown in upper case bold letters, the nucleotide sequences in the exon are in lower case bold letters, nucleotides in the introns are in lower case.

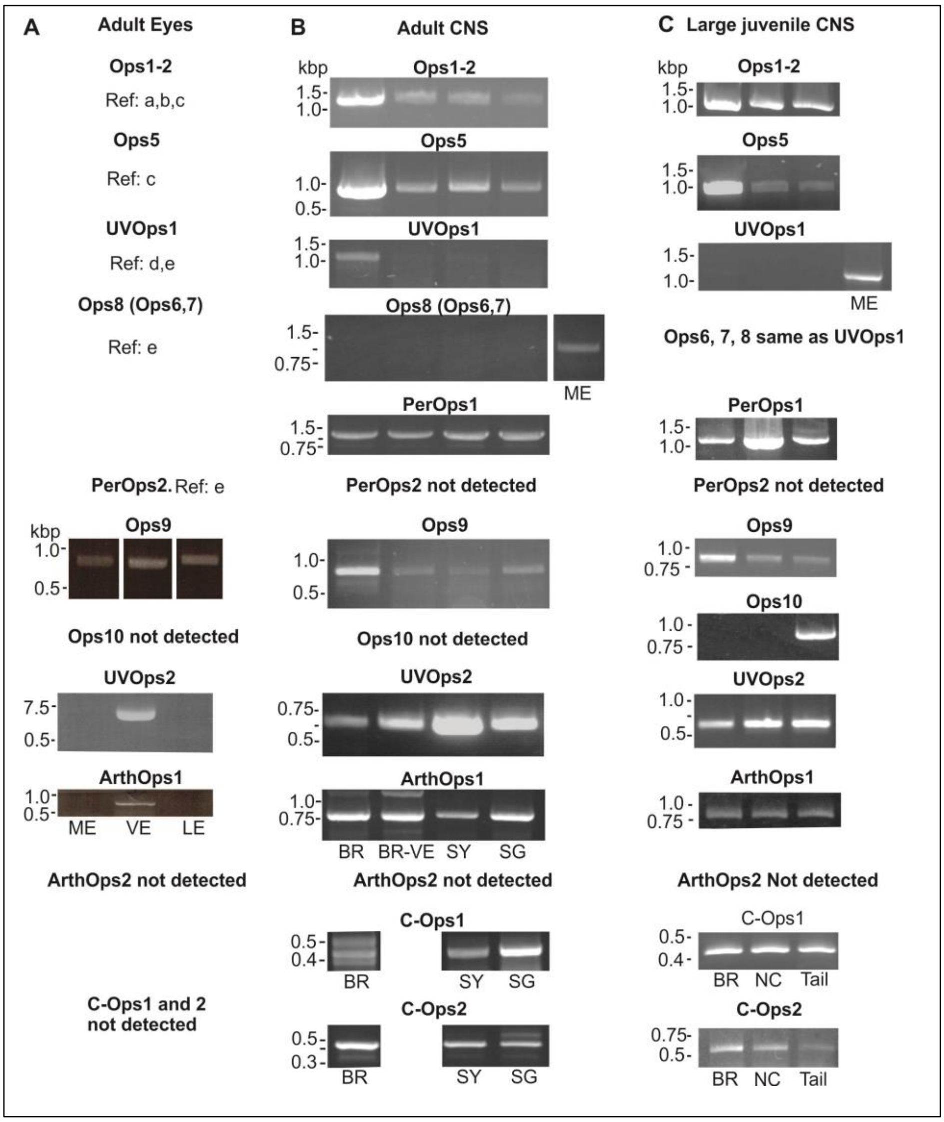

Supplemental Figure 8. Representative results of PCR screens for opsin transcripts in cDNA prepared from A. adult *Limulus* eyes and B. adult *Limulus* CNS and C. the CNS and tail of large juvenile *Limulus*. We screened for opsins in at least two different cDNA preparations of each tissue. The primers used in screens conducted in the current study and the sizes of the anticipated products are listed in Supplemental Table 3. Products obtained in the PCR amplifications were separated on agarose gels. Photographs of the PCR products are shown. The opsin transcript we assayed for is given at the top of each image, the locations of kbp markers run on the same gel are shown to the left to indicate the approximate size of each product, and the tissue from which the cDNA was prepared is listed below each series. In screens for transcripts encoding LpOps 6, 7 and 8 and LpUVOps1 in CNS tissues, cDNA from median eye was included as a positive control. Screens for some opsins in eyes were reported previously and these reports are referenced. ME, median eye; VE, ventral larval eye; LE, lateral eye; BR, brain; BR-VE, brain from which the anterior quarter had been removed to reduce contamination by adhering ventral photoreceptors; SY, synganglion; SG, segmental ganglia; NC, juvenile nerve cord which included the synganglion and segmental ganglia. a. Smith et al., 1993; b. Dalal et al., 2003; c. Katti et al., 2010; d. Battelle et al., 2015; e. Battelle et al., 2014.
