# Supplementary material for: Opsin Repertoire and Expression Patterns in Horseshoe Crabs: Evidence from the Genome of Limulus polyphemus (Arthropoda: Chelicerata)
Source: Genome Biol Evol. 2016 Apr 29;8(5):1571–89. doi: 10.1093/gbe/evw100 (PMC4898813; doi:10.1093/gbe/evw100)
Supplement: Supplementary Data [file supp_evw100_suppl_data.zip › GBE Supplemental Tables 1-4 03-24-2016.docx]

**Supplemental Table 1.** Primers used to clone *Limulus* opsins

| Primer | Sequence 5′ - 3′ |
| --- | --- |
| LpUVOps2 F1 | CAGTTGTACGGCTTTCTGGGTGG |
| F2 | ACGTGAACAGATATGTTCCCGAGGGC |
| F6 | GCTGATCTCGAACATGCCAGAATT CACA |
| R1 | AATTCGGATTTGAAGCGTGGGTGG |
| R2 | GCCCTCGGGAACATATCTGTTCACGT |
| R4 | CGAAAACACACCCATTTCGAGTGG C |
| LpOps9 F1 | CCCTTAGGCTGTCAGCTGTATGGG |
| F2 | ATCACGATAATGACTGATTCGACAGTACTAACAAATTGGAC |
| R1 | GAACGCTTTCGTTTTGTGTTCTTGTTCGTAATATGTA |
| R2 | GAACGCTTTCGTTTTGTGTTCTTGTTCGTAATATGTA |
| LpOps10 F1 | ATGTACGGCTTTATTGGAGGATT |
| F6 | AATGCCACAGTTGTAAGTGCT TTGGATAAATGG |
| R1 | AACGAGGA GAGATAACC CATACA C |
| R5 | AGGGAGCCATGGAAATTTTTTTTTAGTTCCTTCTTAAAAC |
| LpArthOps1 F1 | GGTATC GTCGGCTACGAACGCCA |
| F3 | CACGCACCCCAGTTAATCGAGCTT |
| F4 | ATGTCATTGTGTTTAGATGCCGAAGACTTTATAAGAGC |
| R3 | ACGAGTGCA GGCGCAGAATTC |
| R4 | CTGGCGTTCGTAGCCGACGATACC |
| R5 | AAGCTCGATTAACTGGGGTGCGTG |
| R6 | TTCTGGTTCAGTGACACTGTTTTCATGGAT C |
| LpArthOps2 F1 | GTACCGTCGACTACGAACACC GG |
| F4 | ATGTCGCTTTGTTTTGATGCTCAAAATTTTGTAAGG |
| R3 | GCGAATACAAGAGCAGAACCGACG |
| R6 | TCAGTAGTAGTGTATTATCTTCCGGTCACAGTAATAGC |
| LpCOps1 F1 | ATGATCTTATCACGTGTAGGAATGACAGC |
| F6 | TTCATCGTCATTTGTACGTGGCTCTATTCG |
| R1 | CGGAT GTATACGACACAACTTTTAGCAAAG |
| R10 | CCTCCTCAAGAAAACTCTGTGTTCGGTC |
| LpCOps2 F2 | GGAACTTTGACAGCCATAGCAGTGG |
| F12 | AACCTGGTTGTCATTTCTGTTATTCTTCGGAAC |
| R2 | TAGACATTGGATTATAAACAACGCTACTCTTG |
| R4 | GCGAATATGGCGGGACTAACAGTAGAC |
|  |  |

**Supplemental Table 2** Scorpion opsins mined from the scorpion genome using *Limulus* opsins as queries in tBLASTn. The contig on which the sequences are located and the most closely related *Limulus* opsins are shown.

>contig346362 13097-13423 LpCOPs-like TGMTAIGTVTAIAIERYVILSRHYQSSGLTREKALLCISCIWIYALSLSLPPLFGWNRYIIETPGIACSVDWETTSLVNTSYIIYIFVLGFFAPIFLMVFCYGKVIYIVYKVSLKLIFFLLQAPKITKQSKKAKTERRVTMMAIAMVICTLTAWTPYAVVSLLVTLGFSHFIGPISSIVPAIFAKSSVIYNPIVYFFLNPQVWLNMLACVAK

>con80550-2 2718-3512 5 prime end and 2426-2674 3 prime end LpOps1-like DSVPDDMLYMIDEHWYKFPPINPLWHSLLGIAMIVLGIISIAGNGMVIYLMITTKNLRTPTNLLIVNLAFSDFCMMAFMMPTMAANCFAETWILGPFMCEIYGMAGSLFGCGSIWTMVMISLDRYNVIVRGMAAGPLSTKKAVLMIMFVWTWTITWTLLPFFGWNRYVPEGNMTSCTLDYLNREWHSASYVVIYGCAVYFTPLLTMIYCYFFIVRAVAQHEKTLREQAKKMNVASLRANTDQQKQSAEIRLAKVGNYYTLEQIAMMTVGLWFMAWTPYLSIAWSGIFNNRKHLTPLATIWGSVFAKANACYNPIVYGISHPKYRAALYLKFPALSCTSETDISDVKSE

>Contig256210 LpPerOps-like
FLNLFLNLGLLGTTGNGIIIYTFLRFRIMLSPTTSLLVNLAVADLGICIFGFPFSSSSSFANRINLFILFRWLFGEGGCQWYAFTGFFFGSAHIITLVFLGLDRYLILDRITISKFIILDHFFMIIVIWIYALFWSVMPLLGWGRYNIEPSLTACTIDWRHNDSSYKSFICTYFIFGYVIPCILIAVTYLVTINRIKNNTITVTDIHVRDRWDNEKHMTMVNCF

>Contig291656 LpOps9-10-like
RRNLLIKFFSGCQLYGFIGGLSGTCAILTISAMALERYKIISNPFDANIKLTKSKAFKILIFIWIYALNFCIPPLFGLNRYVPEGYLTSCSFDYLDEKWLHRGFVIVFFVAAWCVPITIICWCYIGIVIKVRNHERTFTQQECRMRIKNKIIKKCSSEVKLAKIAFCLISLWFLAWTPYAIVALIGTFFDRKLLTPLMSMVPALFCKSASVIDPFVYGLTHPKFKIEVYRLLSKIFPCVKIRKRVRSTFRSTKSLESISSDNIEMDYIPQHSPVESIKSVSSENKIEVNKVEVFVIPQVYDIITKNEKIKIKCRRSI

>contig340288 883-2007 LpOps1-like
SHPYQRNATVVDMVPPDMLYMIHEHWYRFAPMNPLWHSLLGIAMIILGIISVVGNGMVIYLMMSTKSLRTPTNLLIVNLAFSDFSMMAFMMPTMAANCFAETWILGAFMCEIYGMAGSLFGCVSIWTMVMITLDRYNVIVRGMSAAPLTHKKAVVLILFVWLWSIAWTIAPMFGWNRYVKICYTNRYVPEGNMTSCTVDYLTKEITYMSYVIVYGCAVYMTPLITMIYSYFFIVRAVATHEKTLREQAKKMNVASLRANADQQKQSAECRLAKVCLIFQTLICVYH

**Supplemental Table 3***. Carcinoscorpius rotundicauda* and *Tachypleus tridentatusm* opsins and opsin fragments recovered from their genomes using *Limulus* opsins as queries in tBLASTn. They are named for the *Limulus* opsin used as query.

>Crops1
MANQLSYSSLGWPYQPNASVVDTMPKEMLYMIHEHWYAFPPMNPLWYSILGMAMIVLGII CVLGNGMVIYLMTTTKSLRTPTNLLVVNLAFSDFCMMAFMMPTMTsNCFAETWILGPFMCEVYGMAGSLFGCASIWSMVMITLDRYNVIVRGMSAAPLTHKKATLLLLFVWIWSGGWTILPFFGWSRYVPEGNLTSCTVDYLTKDWSSASYVILYGIAVYFLPLITMIYCYFFIVHAVADHEKQLREQAKKMNVASLRANADQQKQSAECRLAKVAMMTVGLWFMAWTPYLTIAWAGVFSSGKRLTPLATIWGSVFAKANSCYNPIVYGISHPRYKAALYQRFPSLACGAGESGSDVKSEASATTTMEEKPKNPEA

>CrOps5
MSAESHFIGNATGQRSSGFWTYDPGLSVKDTAPENIKHLIPDHWSKFPAVNPMWHYLLGLIYVVLGIASLTGQSVVLYLFGKTKSLRTPANMLIVNLAFSDFMMMITQFPVFVINCFIGGAWQLGPLLCELTGFAGGLFGYGSIVTLAVVSIDRYNVIVRGFSASPLTHVRAAIFIILIWAWTLGWALPPFFGWGRYVPEGILNSCSFDYLTRDWATISYIMGCWTCEYALPLMVIIYCYIFIVKAVCDHERHLREQAKKMNVASLRSNVDTQKASAEMRIAKVALVNVLLWVVSWTPYAAVAMIGIVG DQMLITPLRSALPALAGKAASVYNPIVYAISHPKFRLAMQKEIPCCCINEPQPQSDTSSEMSTKTSVATVNGSDSTTGESS

>CrOps6
MTDWNFSQSFGHTLWWVSDNEHFLWMRRYGVNVSTTDILPDDMEPLMREYWTQFPVLSPEWFFALGVCMIVLGLISLTGNSVVIYIFITTKSLRTPSNLLVVNLAFSDFMMMFTMMPMMSICCFSQSWILGPLACGVYGAFGSGFGSISIMSMMAIAIDRYRVIVKGLSGERLTYREAFLWICVIWIFVGLWTAAPFFGWNSYVPEGNLTSCSIDMVSHDWSSRSYVIAYGTCVYLLPLLFLSYAYFYIVKTVVEHEYLLRKQARRMNVDRLKPKQPGISVEAKISLVALMTVVLWFVAWTPYAALAFLGSLTNVKLVTPLISIWGAVFAKAGSCYNPIVYAISHPKYQEAFRERIPRLYDFFSCTFCKSSDKDGHQENQHDSTTIEKIISSESSP

>CrOps7 MIEWNHTVSAGLVVSERPDGRYSLWMRRHSDDMMITNLFPHDMEHMIRDHWRQFQALSSKWYFATGVIISILGLIGLLGNFLVIYVFLTTKYLRRPSNMFIINLAFSDFMMMFIIMPMMVVSCFKKKWIFGPLMCEIYGACGSGFGSVSIISMVAIAMNRYRVVVKGLSSEKLTYEQAILWICLIWIYVGLWTSAPFFGWSSYVLEGNFINCSLDSVSHN WNSRSYVIGYCIGMYFLPLCFLTYTYYHIVKTVTKQESVLRKNSRFMNIDQLEPRHSKIT VEAKFSMVALITVVLWFVVWTPYTILILLGSFTSGKLVTPLLSILIAVFAKCASFCDPIV YGISHPKYREALRERMPKLYKIFSCGKCKSKKQNEDPENQHELTKIDKRDSTKESDN

>CrOps8
NPLWFQILGVAMVLLGILCICGNVIIIYIFLTTKSMQTASNVVIVNLAFSDFNCFAEKWALGPFMCEV SLFGCASIWSMVMIIYDSYRIISKDFSVSTMTHNKAALMSIFVWAWSIGWTVPEGNMTSCIFDYLTKDAYSQSYVVIYATAVYYLFLFITIYCYFFVVRAVTDHERSLREQAKKMNVASLRANADQQGTRAEIRLAKIAMFNVGLW FVAWTPYLIISFNGIFSDGAKLTPLATSWGPVFAKANSVYNPIVYGISHPKYRVGLKAKLLWLFCDTDNDENFSTDNNDTSTFITEKMQLPLNIS

>CrOps8short VPEGNMTSCIFDYLTKDAYSQSYVVIYATAVYYLFLFITIYCYFFVVRAVTDHERSLREQAKKMNVASLRANADQQGTRAEIRLAKIAMFNVGLWFVAWTPYLIISFNGIFSDGAKLTPLATSWGPVFAKANSVYNPIVYGISHPKYRVGLKAKLLWLFCDTDNDENFSTDNNDTSTFITEKMQLPLNIS

>CrOps9
RETGDFISTDLTQKRPVDQWREFLSPEIFEIVPDLWLQFPPPTKEAHQVLGAVYTLIMVPGVLGNLLVICLVFS TKALRTPSNILVVNLAISDFLMMTEMPIFIYNSFYQKPAFGVWGCQLYGFMGGLTGTSAIMTIAAMAFERYYSISRSLDLSGRMTRARASAI VICVWIYAFIFSVLPLFHVNRYVPEGYLSSCSFNYLARDLTSRLFVLIFFIAAWCIPLAM IWVSYCGIIFTVRRNQLLFRNPGFQTNHKRLYIQKHRNTEIKLAKIAFTLISLWVISWTP YAMVALFGISFNHELLTPTTSMVPALFCKTASVVDPFLYGLSHPRFKSELKKKIICLDVL NKSAKPKKSFNIPSPSHISNESSFSLQYPSLKQKLEEPLDHQPATISRCHEEIFLTDSQSLPPNILYTDIEHQKHKCFHSVS

>CrOps10
NVTVASSLDKWKTILTSEFLQLVPDHWLQFDPPSDSSHYILGTVYFVVMMTGILGNGTIVWLFCTVKALRTPSVFLV FNLAISDLAMCLMIPSFVYNSFSLGPATGIVGCKMYGVIGGLSGTSAIMTIAAMSMERYYSISKPLGVSGQANWTLIVCAIGFIWFYSSVFSFIPLFGINQYVPEGYLNSCSFDYLSEDLASRRFVLAFFFAAWCIPVSVICVCYLGIGFVVRKHQIYLREQARRMNVQNFASSNQRKTRVQLAKITFCLISLWTLAWTPYAIVALLGVFSRRDLLHPTVSMAPALFCKFASVIDPFVYGLTHPRFKKELKKKF

>CrUvOps1
MVPENLTFRDNQLNVGFLAEQLQQEIHMNGWNAPEDMFINPYWKQFEAPNPFMHYLLGILYTGLMIVACTGNGIVIYVFSRCKTLRTPANLFVVALAVTDFLMMLKTPVFIYNSFHAGPVYGNLGCIIYGTIGAYSGLMSAFCNAVISYDRYRVIACPFSSSKLTNKKALAMLLGIVLYVSPFALLPAFEIWNRYVPEGYLTSCTADYLQHDLNGRSFIFCIWFCAWLIPVIIIFGCYF RIYAAVRDHEKQLHEQAKKMNVENLRTNQNQKDTRGEIRIAKVAFGIIMLFLFSWVPYILVAFIGAFSPKERKLITPLMSMVPALTLKASACFDPFIYAINHPKYRLELQKKLPWLCIHESYSDNASTCSDKTQMSGDTTPTINSDG

>CrUvOps2
ISNMSDLTTSANETRDVEQSDSIDVGLKNSTYSDSVEQWKEILTPEFFELVPDFWLHFPP PSKEAHQTLGVIYTLIMVPGVLGNALIIWLVCSTKTLKTPSNFLVVNLAISDFFMLAKMPIFIYNSFRQKPALGVWgCQLYGFMGGLTGTSAIMTIAAMAFERYYSISRSLDLSGRMTRARASAIVICVWIYAFIF SVLPLFHVNRYVPEGYLSSCSFNYLARDLTSRLFVLIFFIAAWCIPLAMIWVSYCGIIFT VRRNQLLFRNPGFQTNHKRLYIQKHRNTEIKLAKIAFTLISLWVISWTPYAMVALFGISF NHELLTPTTSMVPALFCKTASVVDPFLYGLSHPRFKSELKKKIICLDVLNKSAKPKKSFN IPSPSHISNESSFSLQYPSLKQKLEEPLDHQPATISRCHEEIFLTDSQSLPPNILYTDIE HQKHKCFHSVSCYLNESREKPLEMDVLS

>CrPerOps1_fragment
RWLFNEGGCQWYAFMGFLFGSAHIGVLALLGLDRYLITCRIDFRR KLTYKRYCQMICAVWTYAIFWAVMPLIGWGRYGPEPSITTCTIDWRHNDGSYKSFIIIYFVLGFLVPFLLIAICYYNIARQLSVKPVAPSLRSAVCDQWANERNVTMMCLVIVITFVVSWSPYAIVCLWTVFESPSTVPSLLTLIPPLFAKASTVFNPIIYYLTNPR LRMGIVTVITCSRELPREVIPVSSNPEATSETHESI

>CrPerops2_fragment
RYGPEPSITTCTIDWRHNDGSYKSFIIIYFVLGFLVPFLLIAICYYNIARQLSVKPVAPS LRSAVCDQWANERNVTMCLVIVITFVVSWSPYAIVCLWTVFESPSTVPSLLTLIPPLFAKASTVFNPIIYYLTNPR LRMGIVTVITCSRELPREVIPVSSNPEATSETHE

>CrArthops1
MSLCSDGEDFVRNYIGSFEEMNVSQELRDQSSILLKLLNSSSSVYQGVFSHWCGYSAISS GVHFVVGSFLLLIGVAGIVGNGLVILVLTRYRRLRTPANRLLGNLAVSDLLMSCLHPMASFSSFRHSWQFGKLGCELYGSMCGLFGLVSITTLSVISLERCLVIAMKPWCSGLHINNGKLCKIVAFIWLYSTV CVAPPLFGWGSYVPEGFLTSCSFDYLTRTLVNRAYFVFLYILGFFVPLLVILTSYFTIWKAVFQHEQEMSQIRVNRATSKYVRRSDCKSAEMILYVIGL FLLSWSPYAIVATIGQFWDTNIITPWVSAMPAFFAKMSTIYNPIIYGFSHRQFCACTRHL FTRTQTPALKREEIHS

>CrArthops2
RYRRLRTPANRLIVNLAVSDLMMSFLHFMASYSSFRKSWQFGKLGLGCEFYGSLCGLFGLVSIVTLSAIALERCLIIAIKPWYCSFSITNRKLAKIVAFIWLYCF VCVTPPFLGWGSYVPEGFLTSCSFDYLTRTPTNRAYFFFLFILGFILPLFVIATSYCVIW KTVLQHEREMLQANVSTVSPRFMTRKRSDLKSAVMILCIIGLFLLSWSPYAAIATIGQFF NSSYITPWVSAMPALFAKMSTMYNPIIYGISHRRFCSCIRLLFMKTQIPPPNKKIYMRFS KGISKRDNRQTFLSTARGDYKVSGDVGLQPTNKGRKCYVVMSLDREEKFINTVFSEPQAA HCEKLTYDQRLLEATAHKCQRNLWVRKLLSDSFIYSRSKANGNNDVFLPEYLIKEKPPYK ACFFWYAHKNSSMCYDTTFSCDFWLEALPSIANKEHYVKRPIGGSLPSRCASCYYCDR KIIHCY

>CrCops1
IGTLTAISTERYIIMSRPYGSSKMSPRRSIFIVICTWLYSLSLCLPPFFGWSRYVLEPPG ISCSVDWMTETKNNKSYIIYLFIAGFFLPVFVMVFCYSQIIRMVQKVPRRTGKSHAERAEQRLTLMVATMIISALTAWTPYAVVSLIVALGYSQLIGPLSAVSPAIFAKSCVVYNPIVYFFLNPQVKEAIMETFRRNRPDLQSALQQDISLIATNNYTDVTRRSPVTNNLLSLRSD RTQSFL

>CrCops2
VVISVILRNKQLRSSMNLILLNMSVCDMTISVTGTPLTFVAAVYRRWIFGDLVCEVYGFA MSCAGMTQIGTLTAIAVERYIIMSKPYSSTRMTPKRSGLIIGCTWFYSLGLCLPPLFGWSHYVLEPPGIGCSVDWLSTTKNNVSYIIYLFTVGFAIPVSVMVFCYSHIIWKVRKAPRRTGKSHAERAEQRLTLMVATMIISTLTAWTPYAIVSLIMALGFSHVLGPLSTVS

>TtOps1
SYSSLGWPYQPNASVVDTMPKEMLYMIHEHWYAFPPMNPLWYSILGMAMIFLGIICVLGNGMVIYLMTTTKSLRTPTNLLVVNLAFSDFCMMAFMMPTMTANCFAETWILGPFMCEVYGMAGSLFGCASIWSMVMITLDRYNVIVRGMSAAPLTHKKATLLLLFVWIWSGGWTILPFFGWSR YVYEDNFTSCTVDYLTKDWSSASYVILYGIAVYFLPLITMIYCYFFIVHAVADHEKQLREQAKKMNVASLRANADQQKQSAECRLAKVAMMTVGLWFMAWTPYLTIAWAGVFSSGKRLTPLATIWGSVFAKANSCYNPIVYGISHPRYKAALYQKFPSLACGAGESGSDVKSEASATTTMEEKPKSPEA

>TtOps5
MSAESHFIGNATDQRSSGFWTYDPGLSVRDTAPENIKHLDHWSKFPAVNPMWHYLLGLIYVVLGIASLTGQSVVLYLFGKTKSLRTPANMLIVNLAFSDFMMMITQFPVFVINCFIGGAWQLGPLLCELTGFAGGLFGYGSIVTLAVVSIDRYNVIVRGFSASPLTHVRAAIFIILIWAWTLGWALPPFFGWGRYVPEGILNSCSFDYLTRDWATISYIMGCWTCEYALPLTVIIYCYIFIVKAVCDHERHLREQAKKMNVASLRSNVDTQKASAEMRIAKVALVNVLLWVVSWTPYAAVAMIGIVG DQMLITPLRSALPALAGKAASVYNPIVYAISHPKFRLAMQKEIPCCCINEPQPQSDTSSE MSTKTSVATVNGEDSTTGETSNN

>TtOps6
MTDWNSSQSFGHTLWWVSDNEHFLWMRRYGVNVSTTDILPDDMEPLMREYWTQFPVLSPE WFFALGVCMIVLGLISLTGNSVVIYIFITTKSLRTPSNLLVVNLAFSDFMMMFTMMPMMSICCFSQSWILGPLACGVYGAFGSGFGSISIISMMAIAIDRYRVIVKGLSGERLTYREAFLWICLIWIFVGLWTAAPFFGWNSYVPEGNLTSCSIDMVSHDWSSRSYVIAYGTCVYLLPLLFLSYTYF YIVKTVVEHEYLLRKQARRMNVDRLKPKQPGISVEAKISLVALMTVVLWFVAW TPYAALAFLGSLTNGKLVTPLISIWGAVFAKAGSCYNPIVYAISHPKYQEAFRERLPRLYDFFSCTFCKSSDKDGHQENQHDSTTIEKIILSESSP

>TtOps7- fragment
LRRPSNMFIVNLAFSDFMMMFIIMPMMVVSCFKQKWILGPLMCEIYGACGSGFGSVSIISMIAIAMNRYRVVVKGLSNEKLTYEQAILWICLIWIYVGLWISAPFFGWSSYVLDGNFINCSLDSVSHNWNSRSYVIGYCIGMYFLPLCFLTYTYYHIVKTVTKQESVLRKNS

>TtOps8_C-term fragment
REQAKKMNATSLRANADQQDTRAEIRLAKIAMFNVGLWFVAWTPYLIISFNGIFSDGAKLTPLATIWGSVFAKANSVYNPIVYGISHPKYRVVLKAKLPWLFCNTDNDENFSTDNNDTSTFITEKVQLPLNIST

>TtOps9_C-term fragment
ERYYSISRLLDPRWKMTRLRGTVIAICIWIYSLTFSVMPVFRINQYVPEGYLSGCSFDYIASDLKSRIFVLVFFIAAWCFPVVIICLSYSGIIFIVCKKQFIYRNQEREINFNRFNIQKHKKTEIKLAKLVFALISFWMISWTPYAVVALLGISFNQ QLLTPTISVVPALFCKTASVLDPFLYGLSHPRFKAALREKFSYLQNTGKNTNRKRLFNIHRSQTATLESRESFDHRLSRINRYHEESSETRFQCIPLTVIHPEIDHPQ

>TtOps10
LDKWKTILTSEFLQLVPNHWLQFDPPSDSSHYILGTVYFVVMMTGILGNGTIV WLFCTC VKALRTPSVFLVFNLAISDLVMCSMIPSFVYNSFSLGPATGIVGCKMYGVIGGLSGTSAIMTIAAMSMERYYSISKPLGVSGQANWTLIVCAIAFIWFYSSV FSFIPLFGINQYVPEGYLNSCSFDYLSEDLASRRFVLAFFFAAWCIPVSVICVCYLGIGF VVRKHQLYLREQARRMNVQNFASSNQRKTRVQLAKITFCLISLWTLAWTPYAIVALLGVF SRRDLLHPTVSMAPALFCKFASVIDPFVYGLTHPRFKKELKKKF

>TtUvOps1
MVPENLTFRDNQLNFDFQAEPLQQEIHMNGWNAPEDMFINPYWKQFEAPNPFMHYLLGIL YTGLMIVAC LFIFFRSKTLRTPANLFVVALAVTDFLMMLKTPVFIYNSFHAGPVYGNLGCIIYGTIGAYSGLMSAFCNAVISYDRYRVIACPFSSSKLTSKKALAMLLGIVLY VSPFALLPAFEIWNRYVPEGYLTSCTADYLQHDLNGRSFIFCIWFCAWLIPVIIIFGCYF RIYAAVRDHEKQLREQAKKMNVENIRTNQNQKDTRGEIQIAKVAFGIIMLFLFSWVPYIL VAFIGAFSPKERKLITPLMSMVPALTLKASACFDPFIYAINHPKYRLELQKKLPWLCIHESYSDNASTCSDKTQMSGDTTPTINSDG

>TtUvOps2
DVGLKNSTYSDSVEQWKEVLTPEFFELVPDFWLHFP PPSKEAHQTLGVLYTLIMVPGVLGNALIIWLVCSTKTLRTPSNFLVVNLAISDFFMLAKMPIFIYNSFHQKPALGVWGKCQLYGFVGGMTGTSAIMTIAAMAFERYYSISRSLDLSGRMTRARASAIVICVWIYAFIFSVLPLFHVNRYVPEGYLSSCSFDYLATDLTSRLFVLIFFIAAWCVPLAVIWVSYCGIIFTVRRNKLLFRNPGFQINHKRLYIQKHRNTEIKLAKIAFTLISLWVISWTPYAMVALFGISFNHELLTPTTSMVPALFCKTASVVDPFLYGLSHPRFKSELKKKIICLQVLNESVKPKKSFNIPSPSHISNESSFSLQYPSLKQKLEEPLDHQPSTISRCHEEIFLTDSQSLPSNILYTGIE HQKHKCFHSVSCYLNESREKPLEMDVLS

>TtArthops1
MSLCLDGEDFVRTYIGSSEEMNMSQELRDQSSIFPKLFNSSSSVYQGVFSHWCGYSAISSGVHFVVGSFLLLIGVAGIAGNGLVILVLTRTPANRLLGNLAVSDLLMSCLHPMASFSSFRHSWQFGKLGCELYGSMCGLFGLVSITTLSVISLERCFVIAIKPWCSGLHINNGKLCKIVAFIWLYSTV CVAPPLFGWGSYVPEGFLTSCSFDYLTRTLVNRAYFVFLYILGFF VPLLVILTSYFTIWKAVLQHEQEMSQIRVNSETSRYV--RRSDCKSAEMILCVIGLFLLS

>TtArthops2
RYRRLRTPANRLIVNLAVSDLMMSFLHFMASYSSFRKSWQFGKILGCEFYGSLCGLFGLVSIVTLSAIALERCLVIAIKPWYCSFFITNRKLAKIVAFIWLYCF VCVTPPFLGWGSYVPEGFLTSCSFDYLTRTPTNRAYFFFLFILGFILPLFVIATSYSVIW KTVLQHEREMLQASISTVSPRFMTRKRSDLKSAVMILCIIGLFLLSWSPYAAIATIGQFCNSSYITPWVSAMPALFAKMSTMYNPIIYGISHRRFCSCIRLLFMKTQIPPPNKKIYMRFSKGISRRDNRQTFLSTARGDYRVSGDVDLQPTNKGRKCYVVMSLDQEGKFTNTVSSEPQAA HCENLTHDQRLLEATSHKYQRNLLVRKLLSDSFIYSRSKANGNNDVFLPEYLIKEKPPYN ACFCWYAHKNSSICYDTT FSCDFWLEALPSIANKEHY

>TtPerOps1 Long-- 2 gaps
GILGTVGNGIIITMFIRFRTLVTPTSLLLITLAVSDLGIILGGCQWYAFMGFLFGSAHIGVLALLGLDRYLITCRIDFRKLTYKRYCQMICAVWIYAIFWAVMPLIGWGRRYGPEPSITTCTIDWRHNDGSYKSFIIIYFVLGFLVPFLLIAICYYNIARQLSVKPVAP SLRSAICDQWANERNVTM MCLVIVITFVVSWSPYAIVCLWT SVLTLIPPLFAKASTVFNPIIYYLTNPRLRMGIVTIITCSRELPREVIPVSSNPEATSET HESI

>TtPerOps1 Short one gap
GGCQWYAFMGFLFGSAHIGVLALLGLDRYLITCRIDFRKLTYKRYCQMICAVWIYAIFWAVMPLIGWGRRYGPEPSITTCTIDWRHNDGSYKSFIIIYFVLGFLVPFLLIAICYYNIARQLSVKPVAP SLRSAICDQWANERNVTM MCLVIVITFVVSWSPYAIVCLWT SVLTLIPPLFAKASTVFNPIIYYLTNPRLRMGIVTIITCSRELPREVIPVSSNPEATSET HESI

>TtCops1
IGTLTATSIERYIIMSRPYNSSKMSPRRSCFIVICTWIYSLSLCLPPFFGWSRYVLEPPGISCSVDWMTETRNNKPYIIYLFITGFFLPVFVMIFCYSQIIRRVRKVRKL-HKNSHKVKSNHAHKAQQRLTIMVGIMIICTL TAWTPYAVVSLIVALGYPQLIGPLAAVSPAIFAKSCVVYNPIVYFFLNPQ IQEAIMKTFRRSRPEVQSAPQLDISLIATNNYTDVARRSSVANDLLPLRSDRTQSFL

>TtCOps2
VVIFVILRNKHLQNPFNCILLNMSSCDMTISVMGTPLTFVAAVHRKWIFGDAVCKIYGFGGMTQIGTLTAIVVERYIIMLKLYNSTRVISKRSGLFIASTC YSLNLCLPLLFSWSYYILEPPGIS

**Supplemental Table 4.** Primers used to screen tissues for opsin transcripts

| Primer | Sequence 5′-3′ | Anticipated  product size (kbp) |
| --- | --- | --- |
| LpOps1-2 F8N | GGCTAATCAACTGAGCTACAGTTCTCTGGG | 1127 |
| LpOps1-2 R8N | TGCTTCAGGAATTTTTGGCTT TTCTTCCAT TGT C |  |
| LpOps5 F46 | GTGGTTGGTGGAGCTATGATCCCGG | 900 |
| LpOps5 R43-2 | GGCGTCCACGAGACAACCCAA AGA AG |  |
| UVOps1 F5 | GCAGAGTACCTGGGGATTGTGTCTG | 1066 |
| UVOps1 R4 | GTCCATGGTACCGGCTCTGACTTT |  |
| LpOps6 F6 | ATGACTGGCTGGAATCCCTCACAC | 1100 |
| LpOps6 R5 | TCAGGGAGATGATTCCGGAGAAATTATTTTCTCG |  |
| LpOps7 F2 | ATGGTTGAGTGGAATCATACAGAATCAGCA G | 1184 |
| LpOps7 R2 | GCTTCTGTCGTCGAATTGTTTTTCTCAATT GTT G |  |
| LpOps8 F1 | ATGTTAGACATTATCTCCTCCTCTTCTCCC | 1141 |
| LpOps8 R1b | TTAAGTAGAAATCTTAAAAGGCAGTTG |  |
| LpPerOps1 F6 | GGTGACAGCAGCGGCAGAT | 1140 |
| LpPerOps1 R5 | CATTAATGAGCCACCTAAATACATGCTC |  |
| LpPerOps2 F4 | TTTTCGAGCTTTAACCACGCCAACAAG | 605 |
| LpPerOps2 R6 | AATAAGGGGGAACAGTAAATGGAG |  |
| LpUVOps2 F6 | GCTGATCTCGAACATGCCAGAATT CACA | 650 |
| LpUVOps2 R2 | GCCCTCGGGAACATATCTGTTCACGT |  |
| LpOps9 F4 F | GCTTTAAGGACACCATCCCATATCCTTGTCGTTAAT | 800 |
| LpOps9 R2 R | GAACGCTTTCGTTTTGTGTTCTTGTTCGTAATATGTA |  |
| LpOps10 F1 | ATGTACGGCTTTATTGGAGGATT | 631 |
| LpOps10 R1 | AAC GAG GAT GAG ATA ACC CAT ACA C |  |
| LpArthOps1 F1 | GGTATC GTCGGCTACGAACGCCA | 783 |
| LPArthOps1 R3 | ACGAGTGCA GGCGCAGAATTC |  |
| LpArthOps2 F1 | GTACCGTCGACTACGAACACC GG | 786 |
| LpArthOps2 R3 | GCGAATACAAGAGCAGAACCGACG |  |
| LpCOps1 F2 | CTATTGGAACTCTGACAGCGATATCTACC | 436 |
| LpCOps1 R5 | GACGATCGGATTGTATACGACACAACTTTTAGCAAA G |  |
| LpCOps2 F2 | GGAACTTTGACAGCCATAGCAGTGG | 532 |
| LpCOps2 R2 | TAGACATTGGATTATAAACAACGCTACTCTTG |  |
|  |  |  |
